# Freeform Manufacturing of Plant‐Based Structural Colors for Scalable Photonic and Mechanochromic Devices

**DOI:** 10.1002/adma.202519692

**Published:** 2026-02-06

**Authors:** Xiao Song, Peiqi Niu, Wenxi Gu, Chun Lam Clement Chan, Jiuhong Yi, Xu Liu, Peng Tan, Chon In Haydn Cheong, Qingwen Guan, Dan Fang, Bingpu Zhou, Zi Liang Wu, Ji Liu, Yan Yan Shery Huang, Iek Man Lei

**Affiliations:** ^1^ Department of Electromechanical Engineering University of Macau Macau China; ^2^ Centre For Artificial Intelligence and Robotics University of Macau Macau China; ^3^ Stratingh Institute for Chemistry University of Groningen Groningen Netherlands; ^4^ Department of Chemical and Biological Engineering Princeton University Princeton USA; ^5^ Department of Engineering University of Cambridge Cambridge UK; ^6^ Institute of Applied Physics and Materials Engineering, Joint Key Laboratory of the Ministry of Education University of Macau Macau China; ^7^ Department of Polymer Science and Engineering, Ministry of Education Key Laboratory of Macromolecular Synthesis and Functionalization Zhejiang University Hangzhou China; ^8^ Department of Mechanical and Energy Engineering Southern University of Science and Technology Shenzhen China

**Keywords:** aqueous two‐phase systems, embedded 3D printing, freeform structures, green manufacturing, hydroxypropyl cellulose, structural color

## Abstract

Plant‐based, iridescent, and dynamically tunable structural colored materials are highly attractive for sustainable photonic devices. However, fabricating complex architectures at the decimeter‐scale with optical fidelity using plant‐derived materials remains challenging, limiting their use in photonic devices and adaptive actuation. Here, we introduce an aqueous two‐phase freeform fabrication strategy for vibrantly colored hydroxypropyl cellulose (HPC), where a robust immiscible aqueous environment is developed to preserve HPC cholesteric structures with < 3% shift in peak reflection wavelength over three days, enabling stable processing of large‐scale structural colored materials. Our technique involves a food‐grade support medium with low interfacial tension, allowing for embedded 3D printing of photonic structures and post‐extrusion recovery of the HPC cholesteric domains. Intricate constructs, including interlocking chainmail, with feature sizes down to ∼50 µm and color consistency over lengths exceeding ten centimeters, can be achieved. Additionally, this approach can be utilized to create non‐planar, mechanochromic hydrogel actuators with programmable multicolor designs, as demonstrated in an octopus‐inspired hydrogel actuator and a color‐shifting display for information encryption, camouflage, and human–machine interaction. Our green, freeform manufacturing approach provides new design possibilities for sustainable photonic devices and can be applied to industrially relevant applications.

## Graphical abstract

A green, freeform manufacturing approach that utilizes robust aqueous two‐phase systems to create intricate and scalable photonic structures and non‐planar mechanochromic hydrogel actuators from plant‐based hydroxypropyl cellulose. This approach broadens the structural possibilities of sustainable photonic devices and mechanochromic systems, offering advantages of good optical fidelity during fabrication, freeform design possibilities, scalability potential, and multi‐color designability.

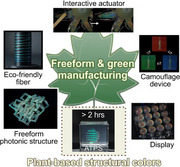

## 1 Introduction

Structural coloration, arising from the reflection of specific wavelengths of light by nano‐ or microstructures, endows biological organisms with exceptional photonic properties, including iridescence, fade resistance, and adaptive coloration [1, 2, 3, 4]. Creating artificial structural colored materials with 3D structures is crucial for applications in biomimicry, adaptive devices, heat management, displays, and sensing [3, 5]. While various printing strategies have been explored, achieving intricate structures with high photonic fidelity via cost‐effective and scalable techniques remains a challenge [5]. For instance, inkjet printing [6, 7] and direct ink writing [8, 9, 10, 11, 12, 13, 14] are typically limited to 2D or simple 3D geometries. Additionally, digital light processing printing has difficulties in processing high‐viscosity inks [15, 16], and two‐photon polymerization printing encounters challenges with high costs and limited scalability potential [17]. Thus, low‐cost methods that enable the creation of freeform photonic constructs at scales larger than a few centimeters while preserving their structural color characteristics during fabrication are highly attractive. Such techniques can innovate the design possibilities for photonic materials.

**FIGURE 1:**
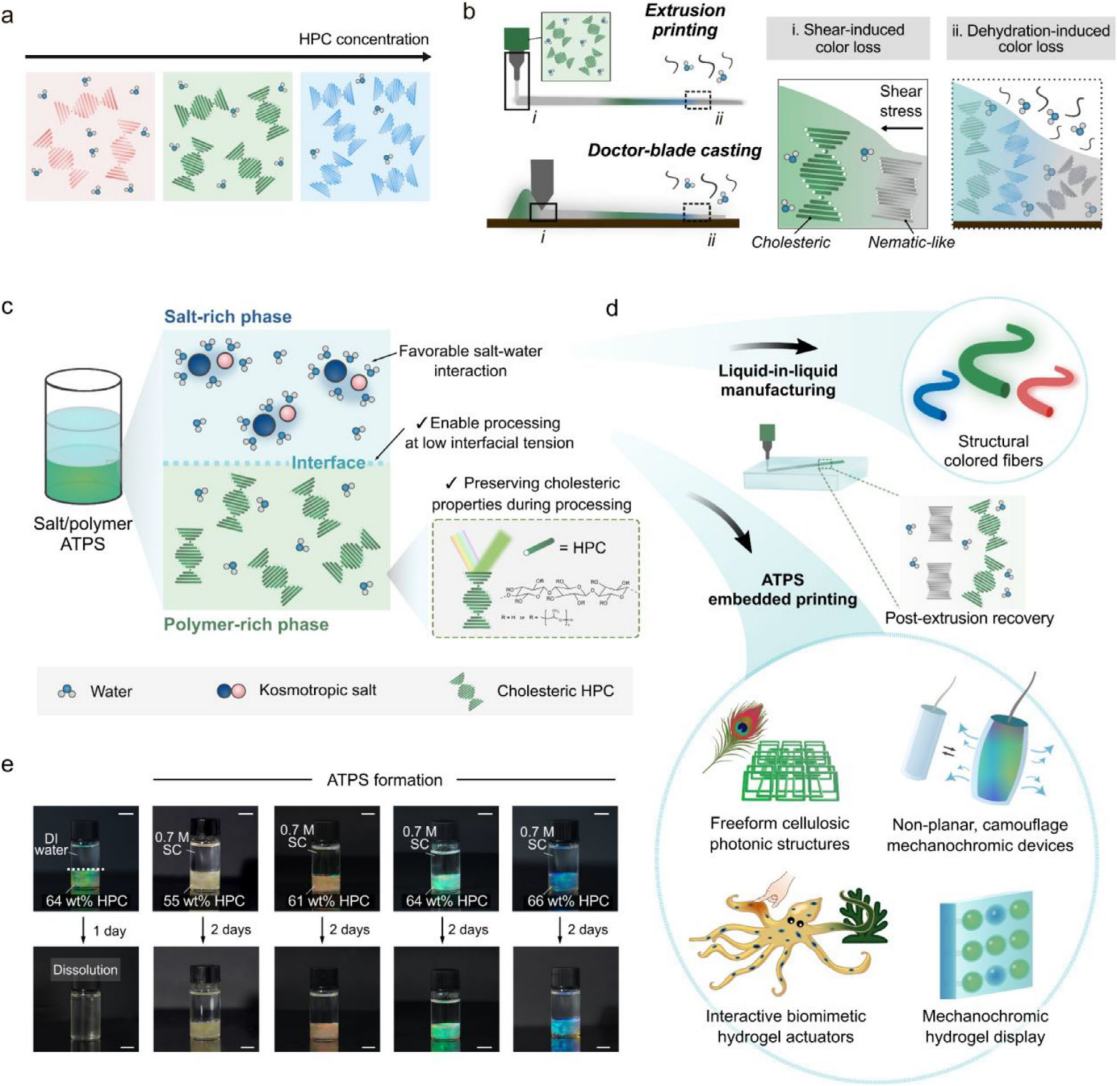
Liquid‐in‐liquid manufacturing of HPC into complex photonic systems. (a) Schematic of HPC cholesteric structures. Increasing HPC concentration leads to a reduction in its cholesteric pitch, causing a blue shift in its characteristic color. (b) Schematic showing the structural color loss issues of HPC during manufacturing processes, such as extrusion printing and doctor blade casting. (c) Schematic illustrating the formation of ATPS between cholesteric HPC and a kosmotropic salt solution. (d) A schematic showing ATPSs enables color preservation and provides low interfacial tension for fabricating complex HPC photonics systems. (e) Photographs showing stable ATPSs formed between 0.7 M sodium citrate (SC) and 55, 61, 64, and 66 wt.% HPC, with no noticeable changes in color after 2 days. In contrast, the 64 wt.% HPC in deionised (DI) water lost its structural color due to dissolution. Scale bars, 5 mm.

Hydroxypropyl cellulose (HPC), a low‐cost and biodegradable cellulose ether, is a sustainable plant‐based feedstock capable of producing structural colors at scale. HPC is non‐toxic and has established use in the pharmaceutical and food industries. Different from cellulose nanocrystals (CNC), which are restricted to film‐like structures due to their evaporation‐induced cholesteric self‐assembly processes, HPC can achieve volumetric structural coloration [18, 19] (Table S1). When dissolved in water at concentrations above 40 wt.%, HPC self‐assembles into right‐handed cholesteric mesophases [20]. Vivid structural color spanning from red to blue can be generated at concentrations of 60–70 wt.% through Bragg‐like reflection [18] (Figure 1a). Despite the promising potential of HPC in sustainable 3D printing [9, 10, 21], sensors [22, 23, 24], mechanochromic devices [23, 25, 26], anti‐counterfeiting [27], and photonic pigments [28], manufacturing complex photonic structures and functional devices with HPC remains challenging. This is primarily due to their high viscosity, viscous behavior (loss modulus, *G*′′ > storage modulus, *G*′), and the loss of structural color caused by the induced shear stress and water evaporation during fabrication [10, 21, 26] (Figure 1b). During fabrication, the shear stress causes nematic‐like alignment, resulting in color loss, which requires a relaxation period for the recovery of the cholesteric structure and color [29]. When processing HPC in air, the unavoidable dehydration can alter its concentration, leading to a colorless appearance. Furthermore, similar to the challenges faced by other structural colored materials, current manufacturing strategies of HPC, such as coating [26], microfluidics [30, 31], injection molding [32], and in‐air extrusion printing [9, 10, 21], can only produce film‐like or simple 3D structures (see Table S1 for comparison).

Here, we propose a freeform manufacturing strategy for cholesteric HPC that utilizes robust liquid‐in‐liquid aqueous two‐phase systems (ATPSs) to enable color preservation during fabrication (Figure 1c). ATPSs are emerging techniques for structuring soft materials, with advantages of ultra‐low interfacial tension and partitioning effects [33, 34]. Our ATPS strategy is based on the immiscibility between cholesteric HPC solutions and a kosmotropic salt solution. The low interfacial tension enables the production of eco‐friendly structural colored fibers with a diameter of 250 µm over meter‐long scales. By integrating ATPS with embedded 3D printing, post‐extrusion recovery of cholesteric domains and vibrantly colored intricate photonic structures can be realized in a food‐grade support bath, such as an interlocking chainmail, with attainable feature sizes as small as ∼50 µm. Our technique supports the manufacture of decimeter‐scale photonic constructs that require prolonged printing, eliminating dehydration‐induced color loss. Additionally, our approach can be extended to create non‐planar mechanochromic hydrogel actuators with multi‐color designability. A series of non‐planar mechanochromic actuators was demonstrated, including a hydrogel actuator for camouflage, an octopus‐mimetic actuator that exhibits human‐robot interaction capabilities, and a color‐changing display for information displaying. This study highlights the advantages of ATPS and embedded printing for processing sustainable photonic materials, which broaden their structural possibilities and offer promising potential for industrial‐scale applications at low cost.

## 2 Results and Discussion

### 2.1 Robust ATPS for Processing HPC With Stable Color

**FIGURE 2:**
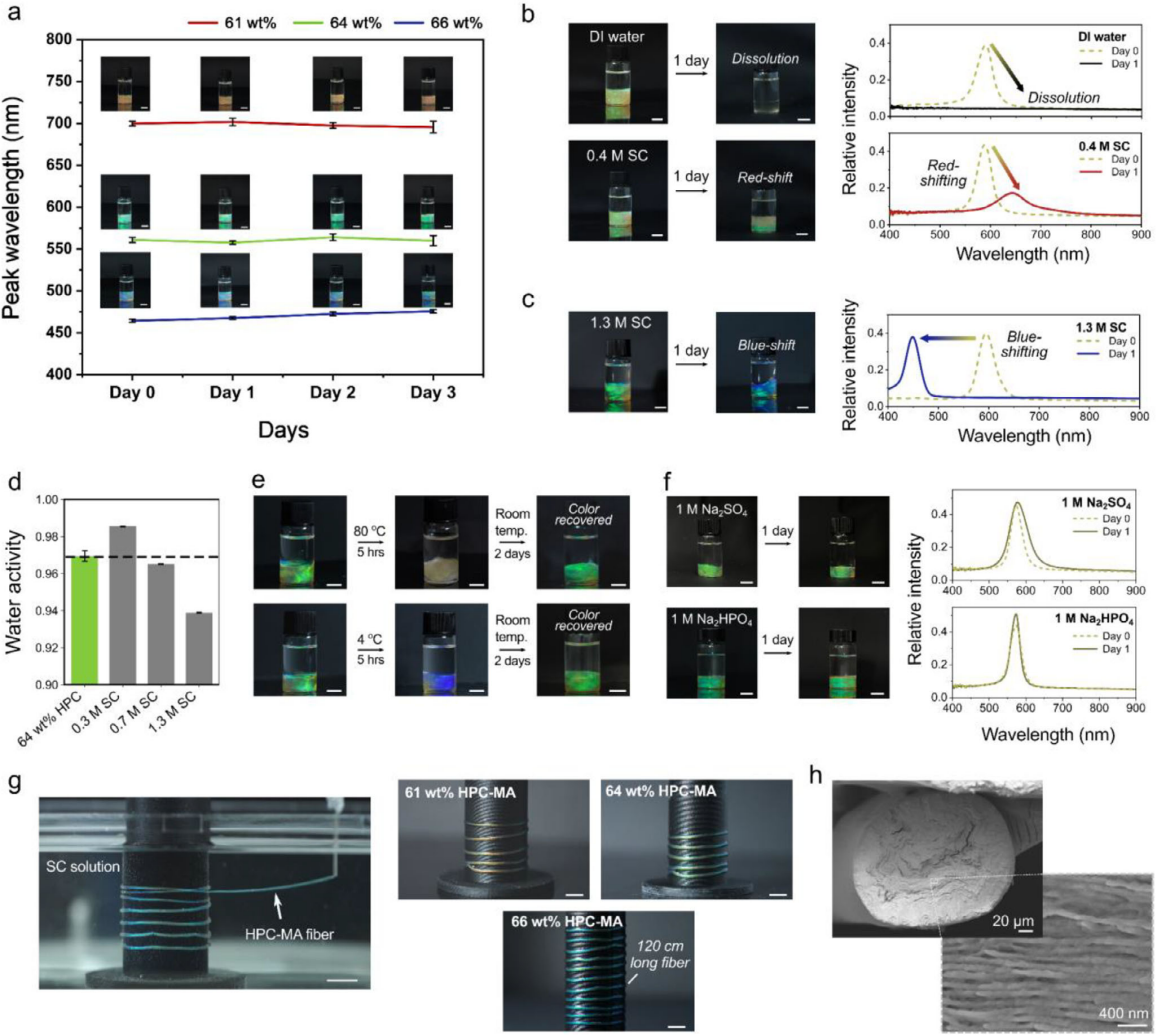
Robust stability of the ATPS and liquid‐in‐liquid manufacture of structural colored fibers. (a) Peak reflection wavelengths of the 61, 64, and 66 wt.% HPC‐rich phases in the ATPSs over three days. Inset photographs illustrate their appearances at one‐day intervals. (b, c) Photographs and reflection spectra indicating that ATPS failed to form with (b) low SC concentrations and (c) excessive SC concentrations. (d) Water activity for 64 wt.% HPC and 0.3 M – 1.3 M SC solutions. (e) Thermal stability of the ATPS composed of 64 wt.% HPC and 0.7 M SC. (f) Stable ATPSs formed between 64 wt.% HPC and other kosmostropic salt solutions, including 1 M sodium sulfate (Na_2_SO_4_) and 1 M disodium phosphate (Na_2_HPO_4_). (g) Red, green, and blue structural color fibers were produced via low interfacial tension liquid‐in‐liquid processing with different concentrations of HPC‐MA. (h) SEM images of an HPC‐MA fiber with a diameter of 250 µm. Data in (a) are means ± SD, *n* = 3 independent samples. Scale bars in (a—c and e—g), 5 mm.

Previous studies have reported the successful use of ATPSs in creating freeform biological constructs [35, 36], ultra‐thin structures [37], and reconfigurable hydrogel systems [34], however, their potential for processing photonic materials remains unexplored. Here, we established a stable ATPS composed of a kosmotropic salt solution as the salt‐rich phase and cholesteric hydroxypropyl cellulose (HPC) as the polymer‐rich phase for manufacturing structural colored constructs with color fidelity (Figure 1d). Sodium citrate (SC) was chosen as the kosmotropic salt due to its non‐toxic, edible properties, and common use as a food additive. The kosmotropic salt solution facilitates ATPS formation through its favorable interaction with water molecules [38, 39] (Figure 1c). As shown in Figure 1e, ATPSs consisting of 0.7 M SC and HPC solutions of 55–66 wt.% exhibited a stable partitioning effect. In particular, the 61, 64, and 66 wt.% cholesteric HPC solutions, corresponding to red, green, and blue colors, preserved their structural colors with less than 3% change in peak reflection wavelengths over 3 days (Figure 2a), and less than 7.5% over 13 days (Figure S1a–d). Additionally, the peak intensity and full width at half maximum (FWHM) of the reflection wavelengths fluctuated by less than 12% and 22 nm, respectively, over the 13‐day period (Figure S1e). Since the color of HPC is sensitive to its concentration, the preserved color indicates that water transport between the phases was effectively inhibited, resulting in no substantial change in the HPC cholesteric structures. To understand the dynamics of water transport between the phases, we measured water activity, which provides insights into the free water condition. The results indicate that the 0.7 M SC solution has water activity similar to that of the cholesteric HPC (Figure 2d; Figure S2), further confirming that no driving force for water transport between the two phases.

In contrast, inappropriate SC concentrations led to significant changes in structural color (Figure S3). When the salt‐rich phase contained no SC or was at lower concentrations, the two phases became miscible, resulting in a rapid red shift in the HPC (Figure 2b). Conversely, excessive SC concentration caused a blue shift in the HPC phase (Figure 2c). This phenomenon is attributed to the increased salting‐out strength, which enhances the hydrophobic interactions of the HPC and decreases their cholesteric pitch. Water activity measurements also indicate that the 1.3 M SC exhibits lower water activity than 64 wt.% HPC (Figure 2d). This difference promotes the transport of free water from the HPC phase to the SC phase, resulting in a blue shift of color.

The partitioning effect in ATPS is influenced by factors such as concentration and temperature [38]. We examined the recoverability of the salt/HPC ATPS after thermal treatment (Figure 2e; Figure S5). When heated to 80°C for < 5 hrs, the 64 wt.% HPC phase exhibited a red shift in structural color and became cloudy, while a blue shift occurred when cooled to 4°C. Remarkably, the HPC structural color and intensity can be fully restored within 2 days when returned to room temperature after thermal treatment. This indicates the robust stability of the ATPS against environmental conditions and its potential for processing cholesteric HPC at extreme temperatures. It should be noted that similar ATPS systems can be established with other kosmotropic salts, such as 1.0 M sodium sulfate, 1.0 M disodium phosphate, 0.8 M potassium citrate, and 1.8 M magnesium sulfate for 61 – 66 wt.% HPC (Figure 2f; Figure S6). These solutions have similar water activity to the cholesteric HPC (Figure S2). To demonstrate the possible utilization of our ATPSs, we produced photonic droplets and fibers with vibrant structural colors using aqueous two‐phase fluidic and wet spinning methods (Figure S7; Figure 2g). Using a photo‐crosslinkable HPC, such as methacrylate‐functionalized hydroxypropyl cellulose (HPC‐MA) produced according to our previously established protocol [21], continuous meter‐long photonic fibers can be produced with potential applications in eco‐friendly textiles (Figure 2g). This process takes advantage of the low interfacial tension of the ATPS to enable structural color fibers with fine diameters (i.e., 250 µm without parameter optimization, Figure 2h), eliminating challenges of discontinuous fibers due to Plateau‐Rayleigh instability, which are often encountered in traditional in‐air extrusion and fiber drawing techniques [40, 41].

### 2.2 ATPS Embedded 3D Printing of Sustainable Freeform, Decimeter‐Scale Photonic Structures

**FIGURE 3:**
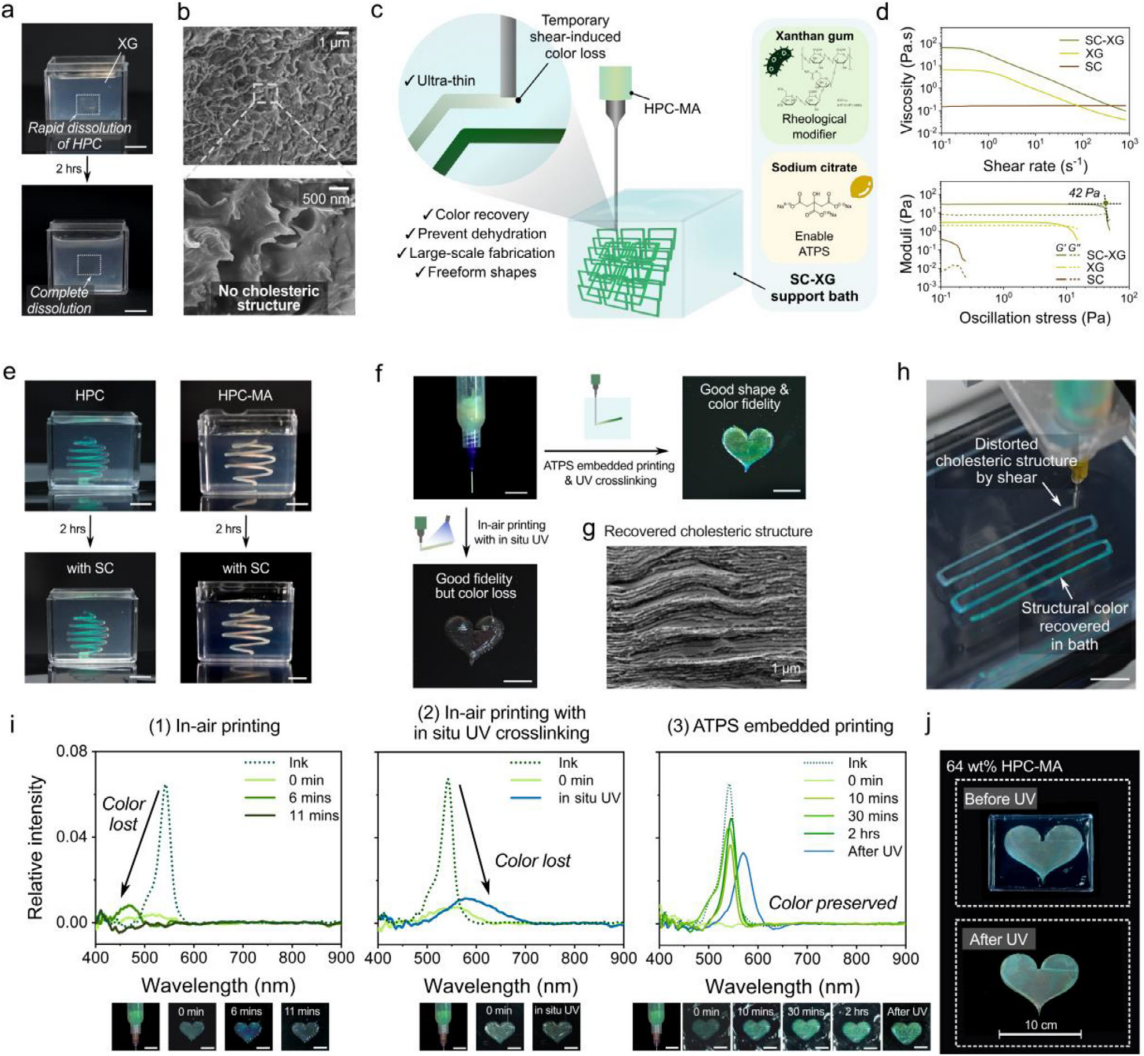
ATPS embedded 3D printing of cellulosic photonic structures with color fidelity. (a) Dissolution problem of a 64 wt.% HPC ink in a 1.5 w/v% XG aqueous bath without adding SC. (b) SEM images revealing the loss of the cholesteric structures of HPC‐MA when embedded and printed in a 1.5 w/v% XG aqueous bath. (c) Schematic highlighting the advantages of ATPS embedded printing for photonic constructs. (d) Apparent viscosity of SC‐XG (1.5 w/v% XG in 0.7 M SC), 1.5 w/v% XG, and 0.7 M SC as a function of shear rate at 25°C. Shear storage moduli (*G^′^*) and loss moduli (*G^″^*) as a function of oscillation stress at 25°C. (e) Stable ATPSs formed between HPC‐based inks (i.e., 64 wt.% HPC and 61 wt.% HPC‐MA) and SC‐XG baths. (f) Comparison showing that ATPS embedded printing enables better color and shape fidelity than in‐air printing. (g) SEM images showing the recovered cholesteric structure of an HPC‐MA filament after 10 min recovery in the SC‐XG bath. (h) Photograph illustrating the structural color loss of the HPC ink after extrusion and its recovery in the bath. (i) Comparison of the changes in the reflection wavelength for (1) in‐air printing, (2) in‐air printing with in situ UV crosslinking, and (3) ATPS embedded printing. The intensity difference between the ink and the printed construct after 30 min of recovery for ATPS embedded printing is due to differences in sample thickness. (j) Large‐scale heart‐shaped construct with preserved structural color. Scale bars in (a), (e), (f), (h), and (i), 10 mm. The inks used in (b, f—j) were 64 wt.% HPC‐MA.

Embedded 3D printing allows for the manufacture of complex freeform structures using non‐self‐supporting inks by performing printing in a yield stress support bath [42]. This technique has displayed promising applications in soft robotics [43], flexible electronics [44, 45], and tissue engineering [46, 47]. However, its potential for constructing structural colored materials has yet to be investigated, primarily due to the ink‐bath interaction that affects their ordered nanostructures. To exemplify the issue, we printed a cholesteric HPC ink in a representative aqueous support bath composed of 1.5 w/v% xanthan gum (XG) (Figure 3a). After deposition, the HPC ink immediately lost its color and became dissolved due to miscibility. Osmotic water transport from the bath to the embedded ink disrupted its cholesteric structure, as evidenced by the SEM images (Figure 3b). Although oil‐based support baths can inhibit water transport between phases, the interfacial tension between the hydrophobic bath and the aqueous HPC ink greatly hinders printability and causes poor adhesion between printed filaments (Figure S8).

We hypothesized that the biphasic behavior between cholesteric HPC and SC solutions can enable freeform all‐aqueous embedded printing of photonic structures, while preserving their structural color characteristics (Figure 3c). To achieve the appropriate rheological properties for embedded printing, we added 1.5 w/v% transparent grade XG to a 0.7 M SC solution to create an immiscible, edible support bath. XG was chosen due to its natural origins, common use as food additives, ease of preparation, and ability to provide the necessary rheological properties. As confirmed by rheological analyses, the SC‐XG support bath exhibits the requisite properties for embedded printing [42, 48]. These include the behavior of Bingham pseudoplastic fluid with a plateau storage modulus ($G_{plateau}^{\prime }$) of 29 Pa and a yield stress (τ_y_) of 42 Pa (Figure 3d), along with thixotropic behavior that allows it to rapidly fluidize and recover when transitioning from high shear to low shear (Figure S9). Importantly, the rheological properties of XG are not weakened by ionic strength, making it well‐suited for the polymer‐salt ATPS embedded printing strategy. In contrast, other commonly used supportive baths, including Carbopol and Laponite XLG, showed remarkable reductions in viscosity and storage modulus when SC was present (Figure S10).

Movie S1 and Figure 3c illustrates the ATPS embedded printing process of an HPC cholesteric ink in a SC‐XG support bath to create freeform photonic structures. When the stress generated from the printing nozzle exceeds the yield stress of the support bath, the support bath fluidizes, allowing smooth nozzle translation without creating crevices. Once the nozzle departs and the applied stress falls below the yield stress, the bath readily recovers, trapping the printed ink in place. Importantly, the formation of ATPS was not affected by the addition of XG. Robust ATPSs were similarly formed between the SC‐XG baths and different concentrations of cholesteric HPC inks and their derivatives, such as HPC‐MA (Figure 3e; Figures S2 and S11 and S12). The structural colors of all printed inks remained stable for at least 2 hrs in the baths. The XG concentration in the bath was optimized at 1.5 w/v%, which enables good print fidelity and effectively resists gravity‐driven sagging (Figure S13).

Our ATPS embedded printing strategy offers substantial advantages over conventional in‐air printing for fabricating photonic structures, as highlighted in Figure 3c. To illustrate its advantages, we employed HPC‐MA inks as crosslinkable inks. Figure S14 outlines the procedure, where an HPC‐MA ink was printed into a SC‐XG support bath and subsequently photo‐crosslinked. In‐air printing often encounters issues of color loss due to compressed cholesteric structures caused by water evaporation during printing, resulting in a blue‐shifted or even colorless appearance of the extruded inks (Figures S15a and S16a). For instance, a centimeter‐scale construct can rapidly lose its color within 11 min of processing due to water evaporation in the air (Figure 3i.1). This limitation severely restricts the potential of in‐air printing for large‐scale fabrication. The color loss issue is particularly pronounced when in situ UV crosslinking is incorporated, which is required for maintaining shape fidelity when printing 3D structures [21]. During the extrusion of the HPC‐MA ink, shear stress distorts the cholesteric domains. Consequently, in situ UV crosslinking fixes the distorted alignment before it recovers, resulting in constructs with a cloudy appearance (Figure 3f,i.2; Figure S15b). In contrast, our ATPS embedded printing overcomes the color loss issue by employing an immiscible support bath, allowing for post‐extrusion recovery of the HPC cholesteric domains and its color in the bath, while preventing dehydration and preserving its shape fidelity (Figure 3h; Figure S16b). As shown in Figure 3i.3, the printed HPC‐MA construct can regain its structural color and color intensity after 30 mins in the bath and maintain its original color for at least 2 hrs in the bath. Microstructure analysis indicates that after recovery in the bath, the printed HPC‐MA filament predominantly exhibited long‐range cholesteric structures with uniform alignment and reduced wrinkling (Figure S17d), successfully regaining the cholesteric structure of the ink (Figure 3g; Figure S18). This morphology is markedly different from in‐air printing, which shows intensive wrinkles and highly compressed cholesteric structures attributed to the evaporation and Helfrich‐Hurault effect [49, 50]. Further discussion can be found in Figure S17. After UV crosslinking, the ATPS‐printed HPC‐MA construct exhibits satisfactory print resolution and color quality that closely matches the ink color (Figure 3f). In addition, as the immiscible bath can prevent dehydration of the HPC‐MA construct during extended printing, large‐scale fabrication can be realized. Constructs over 10 cm in length can be facilely fabricated using a prolonged printing time of 6 hr with good color fidelity (Figure 3j).

**FIGURE 4:**
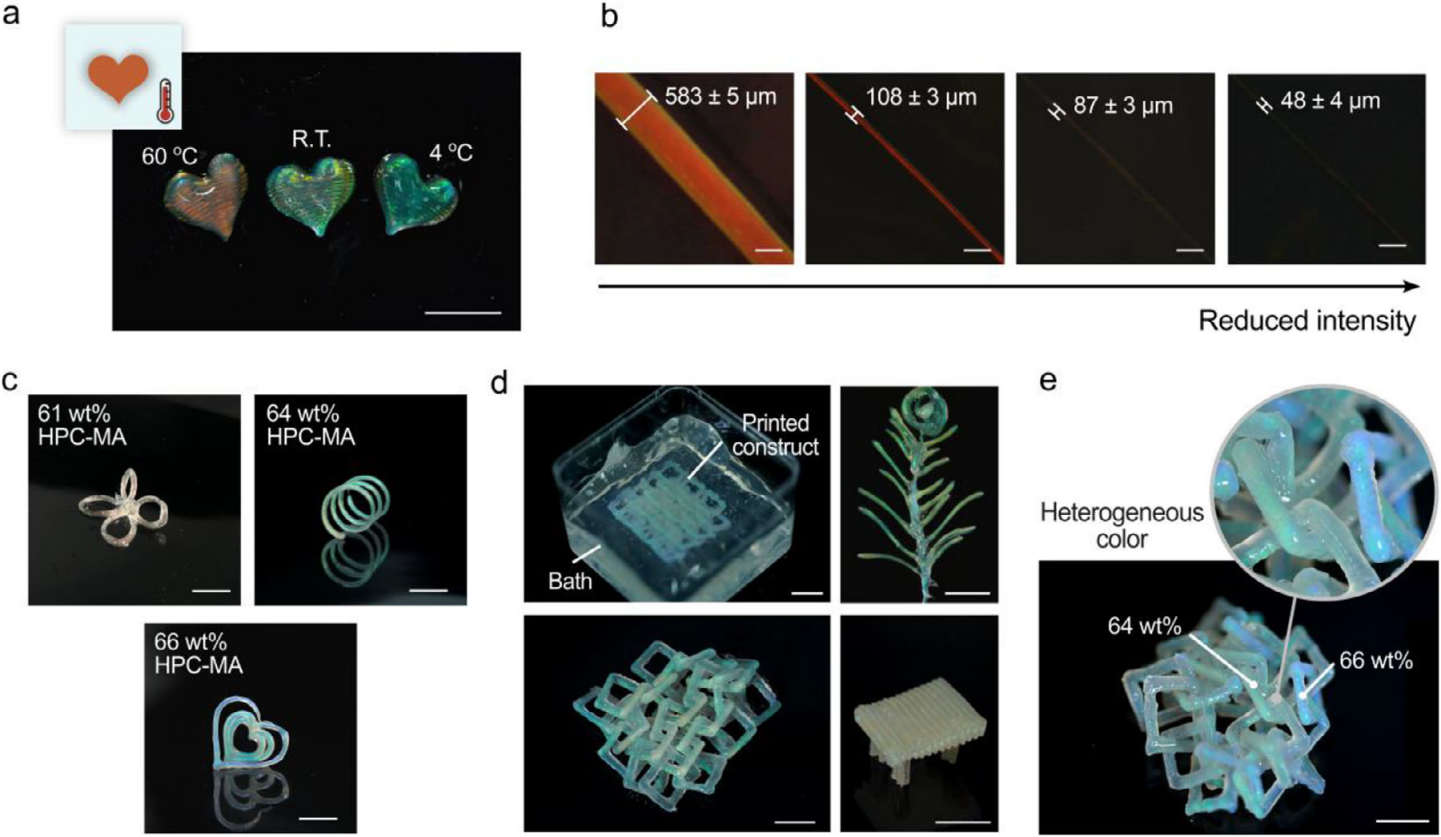
Freeform plant‐based photonic structures with color and intensity tuneability. (a) Cellulosic constructs with varied structural colors fabricated by a single 64 wt.% HPC‐MA feedstock via thermal treatment in bath. The baths containing the HPC‐MA constructs were subjected to thermal treatment for color modulation before UV crosslinking. R.T. = room temperature. (b) Microscopic images of 61 wt.% HPC‐MA filaments produced through ATPS embedded printing, which provides low interfacial tension to achieve ultra‐thin features. (c) Freeform photonic structures produced with HPC‐MA inks at various concentrations via ATPS embedded printing. (d) Intricate freeform cellulosic photonic constructs. The chainmail and the peacock tail feather were produced with 64 wt.% HPC‐MA, whereas the suspended table was produced with 61 wt.% HPC‐MA. (e) Heterogeneous chainmail produced with 64 and 66 wt.% HPC‐MA. Scale bars in (a, c, d, e) = 10 mm, and (b) = 400 µm.

Taking advantage of the thermotropic properties of HPC‐MA and the thermal stability of the ATPS, the support bath containing the printed structure can undergo thermal treatment before UV crosslinking to modulate the cholesteric pitch and the reflected color of the prints. This process enables direct color tuneability with a single ink feedstock (Figure 4a; Figure S19). We attained red‐shifted constructs by heating in a bath at 60°C, and blue‐shifted constructs by cooling to 4°C, followed by UV crosslinking to fix the cholesteric structure. Additionally, optimizing the printing parameters allows for the successful printing of ultra‐fine structurally colored filaments with diameters as small as ∼50 µm, benefiting from the low interfacial tension between the ink and the bath (Figure 4b; Figure S20a). It is worth noting that while even smaller filament diameters are technically feasible with ATPS embedded printing, the resulting structure has reduced color intensity when the diameter is less than 100 µm due to insufficient thickness (Figure S20b). As a result, we can achieve color intensity variation within a single print by varying the thickness of the printed features, which was accomplished by varying the printing speed during printing (Figure S20c). Remarkably, unlike traditional extrusion printing, which is limited to simple and planar photonic geometries [8, 9, 10, 21], our strategy can achieve freeform photonic objects with vivid structural color and suspended features. By varying the HPC‐MA ink concentrations, we produced suspended photonic structures exhibiting red, green, and blue structural colors (Figure 4c; Figure S12). Complex freeform designs that are challenging to produce using traditional 3D printing can be manufactured using our approach, including an interlocking chainmail, a miniature table with suspended features, and a bioinspired peacock tail feather (Figure 4d). Additionally, a chainmail with heterogeneous colors can be produced using multi‐material ATPS embedded printing with different concentrations of HPC‐MA inks (Figure 4e). Although a 6‐h printing time was required for creating the chainmail, the colors remained well‐preserved without dehydration‐induced color loss. Overall, our ATPS embedded printing approach advances the structural complexity of photonic objects achievable with 3D printing.

### 2.3 Printable Mechanochromic Devices with Multi‐Color Designability

Many soft organisms, such as cuttlefish and octopuses, can dynamically change their colors in response to environmental stimuli, protecting themselves from threats. Emulating this dynamic coloration in soft and stretchable hydrogels is appealing for applications in biomimetic soft robots, robotic skins, and camouflage. Although various flexible materials have been developed with color‐changing capabilities, such as cholesteric liquid crystal elastomers [51, 52, 53], photosensitive polymers [54], and mechanophore‐functionalized polymers [55, 56], these materials often involve complex synthesis processes and fail to replicate the high water content and mechanical resilience of biological soft tissues. Therefore, simple fabrication techniques that can create mechanochromic hydrogels with multi‐color designability are highly attractive.

**FIGURE 5:**
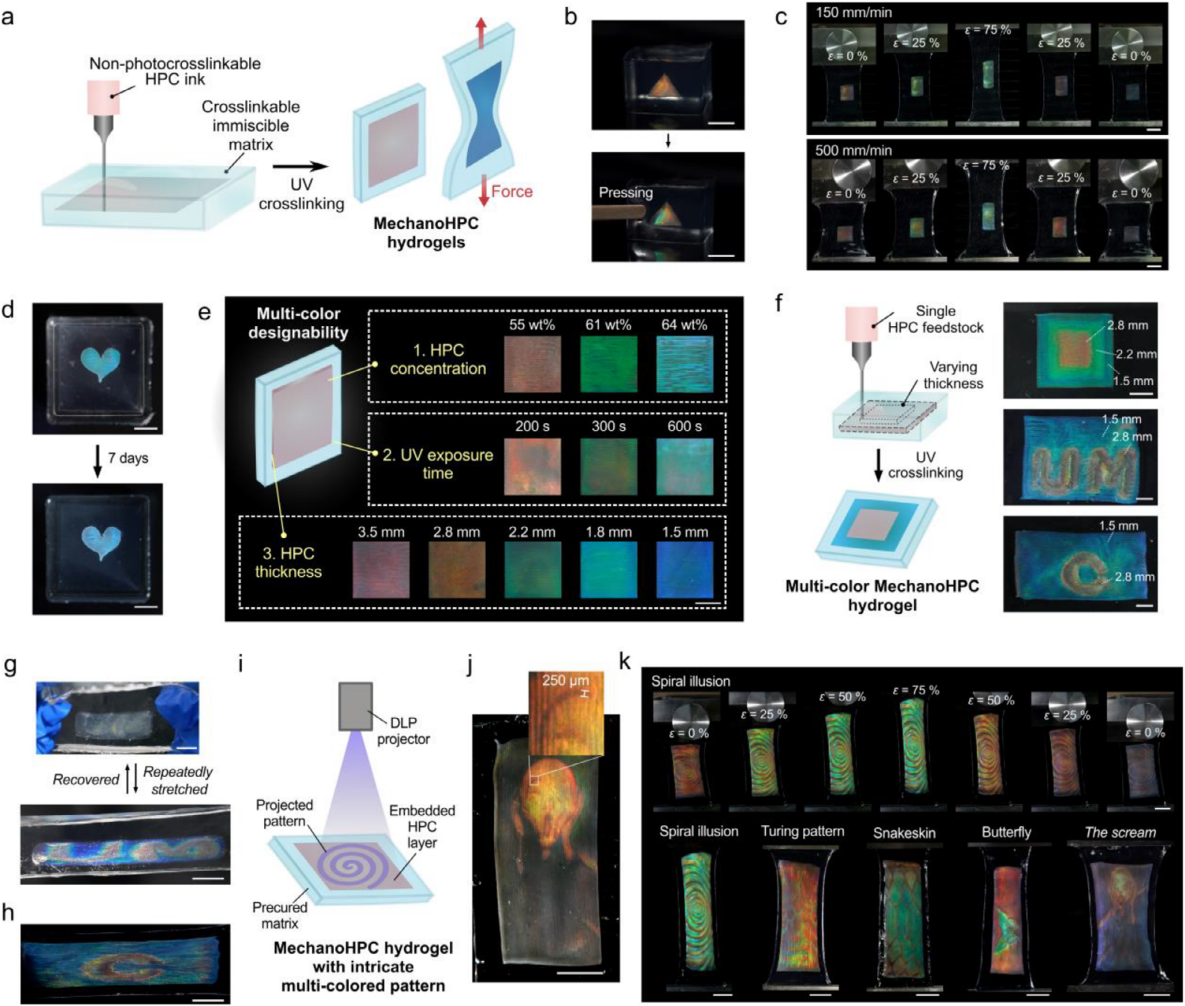
MechanoHPC hydrogels enabled by ATPS embedded printing. (a) Schematic of the fabrication procedure for creating MechanoHPC hydrogels. (b) 3D MechanoHPC hydrogel with pyramid faces. (c) Photographs of the planar MechanoHPC hydrogels when stretched to 75% strain at different deformation rates. (d) Long‐lasting structural color of the MechanoHPC hydrogel when coated with PDMS. (e) Factors for tuning the structural color from red to blue. The UV crosslinking time used in (e.1) was 200 s, and (e.3) was 600 s; the HPC thickness used in (e.1) and (e.2) was 1.8 mm, and the concentration of the HPC inks employed in (e.2) and (e.3) was 55 wt.%. (f) Embedded printing to create MechanoHPC hydrogels, with the encapsulated HPC exhibiting programmable thickness‐dependent structural colors. (g, h) Vibrant and dynamic color responses of the MechanoHPC hydrogels printed with the “UM” and “C” patterns exhibited upon mechanical deformation. (i) Schematic illustrating high‐resolution patterning of structural colors on MechanoHPC hydrogels with the DLP technique. (j) MechanoHPC hydrogel DLP‐printed with the painting of *The Scream*. (k) Photographs of the stretchable MechanoHPC hydrogels DLP‐printed with intricate and multi‐colored patterns. Scale bars in (b–d, f–h and j, k) = 10 mm, and (e) = 5 mm.

The ATPS proposed in this study provides a straightforward approach to fabricating mechanochromic hydrogels with mechanical resilience and structural color designability. By embedded printing a liquid‐state pristine cholesteric HPC ink into a photo‐crosslinkable, immiscible aqueous matrix, we developed mechanochromic HPC hydrogels, referred to as MechanoHPC hydrogels (Figure 5a). The matrix contained SC as the kosmotropic salt, XG as a rheological modifier, acrylamide (AAm) as a crosslinkable monomers, *N,N'*‐methylenebisacrylamide (MBAA) as the covalent crosslinker, and ammonium persulfate (APS) as a photo‐initiator. Importantly, the inclusion of AAm did not compromise the stability of the ATPS, with no noticeable color change in the embedded HPC phase for up to 12 hrs, allowing for extended printing times (Figures S2 and S21a). Additionally, the matrix exhibited Bingham pseudoplastic and thixotropic properties for embedded printing (Figure S21b). After printing, the matrix was UV crosslinked to immobilize the printed HPC materials, which exhibited a blue shift in its structural color, attributed to the matrix shrinkage effect and water loss during crosslinking (Figure S22). 3D freeform HPC constructs can be printed and stably embedded in the matrix with mechanochromic behavior (Figure 5b). In contrast, without kosmotropic salts in the matrix, the structural color of the embedded HPC pattern rapidly faded due to phase miscibility (Figure S21c), similar to the effect discussed in Figures 2b and 3a.

The poly(AAm) network imparts outstanding mechanical resilience and stretchability to MechanoHPC hydrogels, making them suited for repeated use. Figure S23a shows that the tensile stress‐strain curves of the matrix remained stable over 50 cycles with negligible hysteresis. The elongation‐at‐break can range from 120% to 700% by varying the crosslinking time (Figure S23b). Increasing the UV crosslinking time resulted in a stiffer matrix, with Young's modulus increased by 3.7‐fold as crosslinking time increased from 120 to 600 s. To investigate the mechanochromic performance, we fabricated hydrogels embedded with a planar MechanoHPC unit and UV crosslinked for 200 s. When stretched to a strain of 75%, the hydrogel displayed a continuous red‐to‐blue color transition (Figure 5c; Movie S2). The mechanochromism gradually attenuated during continuous tensile cycles, which can be attributed to the shear‐thinning properties of HPC noted in previous literature [25, 57], but the color changes became stabilized after 20 cycles (Figure S24). Improved mechanochromic recovery of HPC can potentially be achieved with recent advances in the design of HPC formulations, such as the HPC‐gelatin formulation [57]. MechanoHPC hydrogels with a color‐changing effect under compression can be similarly produced (Figure S25 and Movie S3). To enable long‐lasting structural coloration, the MechanoHPC hydrogels can be coated with PDMS to prevent dehydration. The structural colors of the hydrogels can persist for more than 7 days with a PDMS coating (Figure 5d).

The structural color of MechanoHPC hydrogels can be broadly controlled by the HPC ink concentration, UV exposure time, and thickness of the embedded HPC (Figure 5e). For instance, increasing the concentration of HPC ink from 55 to 64 wt.% results in a color shift from red to blue (Figure 5e.1). Additionally, altering the UV exposure time of the matrix or the thickness of the embedded HPC allows for red‐to‐blue color tunability using a single 55 wt.% HPC ink feedstock. Specifically, prolonging the UV exposure time from 200 s to 600 s leads to a blue shift in the embedded HPC, attributed to the volumetric shrinkage effect caused by the rapid free radical polymerization of the matrix and temperature‐induced water loss during UV exposure (Figure 5e.2, further discussed in Figure S26). On the other hand, decreasing the thickness of the embedded HPC from 3.5 to 1.5 mm while keeping a constant UV crosslinking time shifted its structural color from red to blue, attributed to the increased compression effect to the HPC layer caused by the polymerization of the matrix (Figure 5e.3; Figure S26). Noteworthily, the colors of the patterns were consistent regardless of their 2D shapes, when produced under the same crosslinking time (Figure S27).

Previously reported HPC mechanochromic devices only exhibited a single color at their non‐stressed states. This limitation arises from the difficulties in spatially controlling the structural colors of liquid‐state HPC solutions in these devices. Our approach allows for spatial control of MechanoHPC hydrogels using a single HPC feedstock, enabling the creation of highly stretchable mechanochromic devices with multi‐color designability. By harnessing the dependence of HPC colors on their thickness within the matrix, we can embedded print HPC patterns with colors spatially mapped to their thickness using a single HPC ink (Figure 5f). Various patterns, such as squares and letters, were created, which displayed multiple eye‐catching colors that changed dynamically upon deformation (Figure 5g,h; Movie S4). Additionally, our fabrication procedure can integrate with the digital light processing (DLP) technique to create intricate, multi‐structural colored patterns on MechanoHPC hydrogels (Figure 5i). These hydrogels were made by embedded printing a cholesteric HPC layer into a photo‐crosslinkable matrix, followed by pre‐curing. Subsequently, a DLP projector with a 385 nm LED was utilized to project a custom UV pattern onto the pre‐cured MechanoHPC hydrogel, enabling spatial control over its crosslinking degree and structural coloration. A patterning resolution of at least 250 µm can be achieved, which can be further enhanced up to the limits of advanced optical lithographic resolution (Figure 5j). As illustrated in Figures 5j,k and Figure S28, this approach enables the creation of various MechanoHPC hydrogels with intricate multi‐colored patterns. These include a rotating spiral illusion pattern, biomimetic patterns, such as snakeskin and Turing patterns, a butterfly pattern, and the painting of *The Scream*. When stretched, all MechanoHPC hydrogels exhibited lively and dynamic colorations (Movie S5). These hydrogels can serve as smart skins for robots, providing sensitive visual responses and rapid biomimetic pattern adjustments for effective camouflage, while offering a sense of softness and a skin‐like touch.

### 2.4 Non‐Planar Pneumatic MechanoHPC Hydrogel Actuators

**FIGURE 6:**
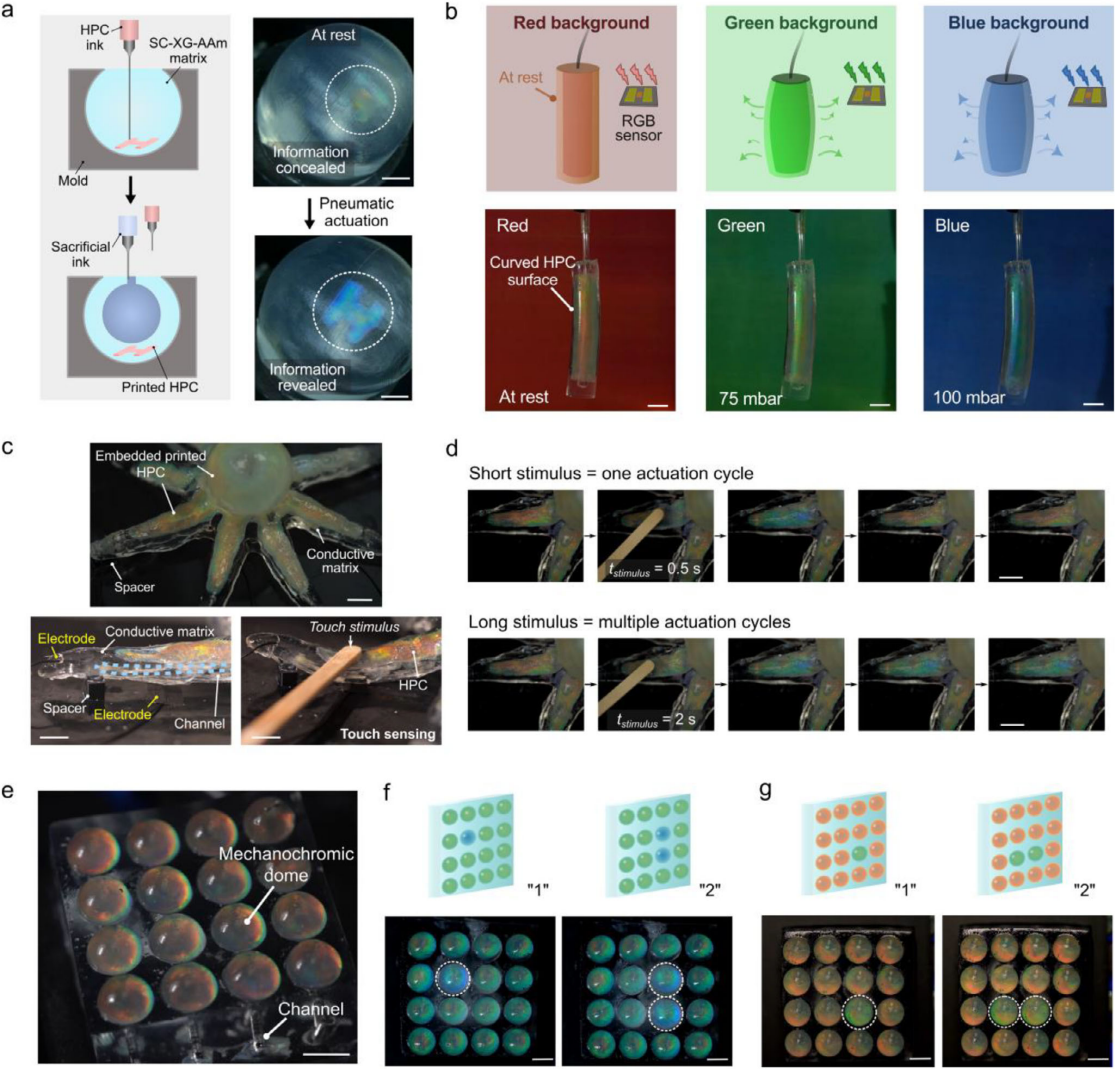
Non‐planar MechanoHPC hydrogel actuators. (a) Multi‐material 3D printing of the HPC ink and sacrificial ink to create an information encryption device. (b) Cylindrical MechanoHPC actuator that can dynamically shift its color to match its surrounding background by pressurization, enabling real‐time camouflage. (c) Bioinspired MechanoHPC octopus actuator. Its spacer design enables tactile sensing and human‐robot interaction with coloring‐changing responses to touch stimuli. (d) The capability of the MechanoHPC octopus actuator to detect touch duration (*t_stimulus_*), triggering different response durations accordingly. (e) MechanoHPC hydrogel displays with mechanochromic domes and pneumatic channels, which can be pressurized for a color‐changing effect. (f, g) Customizable mechanochromic hydrogel displays showing dynamic multi‐color patterns. Displays in (e‐g), which were exhibited in different initial colors, were treated with UV crosslinking for 350, 600, and 450 s, respectively. Scale bars, 10 mm.

Embedded printing enables freeform incorporation of various functional inks, facilitating the integration of multiple functional features into devices [43]. We demonstrate that sacrificial inks can be printed into the matrix precursors of the MechanoHPC hydrogels, enabling actuation capabilities. We fabricated an actuator for information encryption by embedding 3D printing a 55 wt.% HPC ink and a sacrificial ink composed of 35 w/v% Pluronic F127 and 2 w/v% ascorbic acid into a SC‐XG‐AAm precursor matrix within a custom‐designed mold (Figure 6a; Figure S29). After printing, the SC‐XG‐AAm matrix was UV crosslinked, and the fabricated MechanoHPC actuator was removed from the mold. Pluronic F127 is commonly used as a sacrificial ink due to its ability to transition between liquid and gel by varying the temperature. We added ascorbic acid to the sacrificial ink as a radical scavenger, which can prevent photo‐polymerization of the diffused AAm monomers in the printed sacrificial ink region (Figure S30). Using this approach, the sacrificial ink can easily be evacuated from the matrix, resulting in an actuator with a hollow cavity for actuation. As demonstrated in Figure 6a and Movie S6, the actuator revealed letter information through pneumatic actuation, which compressed and blueshifted the HPC layer, while concealing the information when pressure was released.

Existing mechanochromic devices, including cholesteric HPC systems, are predominantly limited to simple planar geometries [22, 26, 58] due to the fabrication challenges. The freeform manufacturing capabilities of our ATPS embedded printing approach allow for fabrication of non‐planar mechanochromic hydrogel devices with tailor‐designed exterior shapes. To demonstrate this capability, we created several non‐planar mechanochromic hydrogel devices. In our first demonstration, we printed a cylindrical MechanoHPC actuator featuring a 360° curved mechanochromic surface and a templated fluidic channel for pressurization (Figure S31a). When coupled with an RGB sensor, the actuator, which appears red at rest, can detect the local background color and instantly change its color homogeneously through pressurization to match the background, achieving camouflage (Figure 6b; Figure S31b and Movie S7). Furthermore, drawing inspiration from blue‐ringed octopuses, which use sudden color changes in their soft skin as a warning to predators when threatened, we created a bioinspired MechanoHPC octopus actuator with tactile sensing and human‐robot interaction capabilities. Figure S32 illustrates its control logic. The arms and head of the MechanoHPC octopus contain non‐planar printed HPC, with each arm embedded with a pneumatic channel fabricated using sacrificial ink (Figure 6c). Additionally, spacers were positioned between the ionically conductive SC‐XG‐AAm arms and the external electrodes for sensing capabilities. When the conductive arm is touched and contacts the external electrode, a voltage signal is detected by an Arduino microcontroller, and the arm automatically responds with a color change by selectively pressurizing the actuation channel for varying durations according to the duration of the touch stimuli (Figure 6d; Movie S8). Finally, we show that soft hydrogel displays with mechanochromic domes can be printed using ATPS embedded printing (Figure 6e; Figure S33). The initial color of the mechanochromic domes is tunable through different fabrication parameters, such as UV crosslinking time (Figure 6e–g). These mechanochromic domes can function as dynamic color‐changing pixels, which experience a reversible blue shift when pressurized. By selectively pressurizing individual domes, multi‐colored patterns can be dynamically displayed (Figure 6f,g; Movie S9). Overall, our ATPS embedded printing approach provides a low‐cost and efficient approach for manufacturing non‐planar sustainable mechanochromic hydrogel actuators with promising applications in camouflage, interactive devices, and display technologies.

## 3 Conclusions

This work presents a freeform manufacturing approach for plant‐based cholesteric hydroxypropyl cellulose (HPC), addressing the challenges of achieving complex photonic structures at the decimeter scale while preserving color properties in traditional 3D printing. Our method utilizes a food‐grade immiscible aqueous bath, uniquely offering the advantages of processing at low interfacial tension, post‐extrusion recovery of cholesteric structures, and preventing dehydration‐induced color loss. HPC color can be preserved with less than 3% change in its peak reflection wavelength over 3 days. Large‐scale (> 10 cm) and freeform sustainable photonic structures, such as an interlocking chainmail, can be produced with excellent shape and color fidelity without requiring rheological modifiers in the inks, and a satisfactory print resolution of < ∼50 µm can be achieved. Additionally, our approach can be extended to create non‐planar mechanochromic hydrogel devices, providing possibilities for programmable multi‐color designs and the integration of other functional inks, such as sacrificial inks for actuation. We developed a series of non‐planar mechanochromic hydrogel actuators, including a hydrogel pneumatic actuator for camouflage, a bioinspired octopus device with human‐robot interaction capabilities, and a pneumatic display with color‐changing pixels. Overall, this work advances the complexity of photonic structures that can be achieved with 3D printing. The food‐grade processing approach ensures the safety and ease of use of our technique. The versatility of ATPS can be leveraged for other structural colored systems, inspiring innovations for sustainable 3D printing, soft robotics, and adaptive devices with promising industrial‐scale potential.

## 4 Experimental Section

### 4.1 Materials

Hydroxypropyl cellulose (HPC, SSL grade, M_w_ = 40,000 g mol^−1^) was purchased from Nippon Soda Co., Ltd. Citric acid trisodium salt (SC), acrylamide (AAm), sodium sulfate, lithium phenyl(2,4,6‐trimethylbenzoyl)phosphinate (LAP), L(+)‐ascorbic acid, poly(ethylene oxide)‐*b*‐poly(propylene oxide)‐*b*‐poly(ethylene oxide) triblock copolymer (Pluronic F127) were obtained from Adamas. Disodium phosphate was purchased from Sigma‐Aldrich. Nigrosine, citric acid, potassium citrate, magnesium sulfate, sodium hydroxide (NaOH), *N,N'*‐methylenebisacrylamide (MBAA), methacrylic anhydride (MA), and ammonium persulfate (APS) were purchased from Aladdin. Transparent grade xanthan gum (XG) was acquired from Taiborui Chemical. The Sylgard 184 silicone elastomer kit was obtained from Dow Corning. Unless otherwise stated, all chemical compounds were used as received, without further modification. All water used in the experiments was deionized water.

### 4.2 Preparation of Salt‐Rich Phases

Three types of salt‐rich phases, which can form stable ATPSs with HPC‐based solutions, were prepared under ambient conditions. Kosmotropic salt solutions, such as 0.7 M SC, were prepared by dissolving specific amounts of kosmotropic salts in deionised water using a magnetic stirrer until complete dissolution. To produce salt‐rich support baths for embedded printing HPC constructs, 1.5 w/v% XG was added to 0.7 M SC solution, and the mixture was stirred at 1000 RPM for at least 5 hrs using an overhead stirrer (DLAB, OS20‐Pro). To prepare photo‐crosslinkable salt‐rich matrices for fabricating MechanoHPC hydrogels, a typical batch involved adding 10 g of AAm, 18.11 g of SC, 50 mg of APS, 100 mg of MBAA, and 1.5 g of XG per 100 g of water. The container with the mixture was then wrapped with aluminum foil and stirred until a homogeneous mixture was obtained. The mixture was kept protected from light exposure prior to printing.

### 4.3 Preparation of HPC Cholesteric Inks

HPC inks were prepared as follows: HPC powder was added to water to create solutions at concentrations of 55, 61, 64, 66, and 67 wt.%. These solutions were mixed for 2 min at 2000 RPM using a planetary mixer (AR100, Thinky Mixer), followed by degassing using centrifugation at 4000 RPM for 90 min. Unless otherwise specified, a 55 wt.% HPC ink supplemented with a trace amount of nigrosine was used for all MechanoHPC devices to enhance the contrast of HPC structural color. Specifically, the formulation contained 9 g of HPC and 100 mg of nigrosine in 11 g of water.

HPC‐MA was synthesized according to our previously established protocol [21]. Specifically, 10 g of HPC powder was dissolved in 500 mL of water, followed by the addition of 40 mL of MA. Subsequently, 59 mL of 5 M NaOH was added dropwise over 30 min while continuously stirring at room temperature. The reaction was allowed to proceed overnight. The resulting solution was dialyzed for at least 3 days and then freeze‐dried to obtain the HPC‐MA product. Inks with concentrations of 61, 64, and 66 wt.% HPC‐MA was prepared by dissolving the appropriate amount of HPC‐MA powder, 1 wt.% LAP (relative to HPC‐MA), and 0.5 wt.% nigrosine in water. This mixture was then mixed in a planetary mixer and centrifuged. Prior to printing, the HPC‐MA inks were stored at 4°C under light‐protected conditions.

### 4.4 Preparation of Sacrificial Inks

The sacrificial inks used for fabricating pneumatic MechanoHPC actuators were prepared by dissolving 70 mg of L(+)‐ascorbic acid in 10 mL of water. 3.5 g of Pluronic F127 was then added to the mixture, which was stirred at 400 RPM using a magnetic stirrer in an ice water bath until dissolved. The ink was loaded into a 10 mL barrel, centrifuged for 2 mins using a planetary mixer (AR‐100, THINKY Mixer), and stored at room temperature prior to printing.

### 4.5 Freeform ATPS Embedded Printing of HPC‐MA Constructs

All 3D printed HPC and HPC‐MA samples were fabricated using a direct ink writing 3D printer (Bio‐Architect SR, Regenovo). The STL files of the printed structures, including models of a heart and chainmail, were obtained from Thingiverse.com (heart box by ToriLeighR, and Fabric of Thyme 2.0 chainmail by Trombo) under the Creative Commons license. The XYZ coordinates that describe the print paths of the one‐stroke structures, illustrated in Figures 3e and 4c and Figures S11 and S12, were generated and converted to G‐code files using a Python script developed in‐house based on the equations of the spherical helix curve, the Butterfly curve, and the Heart curve. Detailed information about the equations used is provided in Table S2. To ensure a smooth print path, the curve was discretized by at least 500 evenly spaced points. The coordinates used for printing the peacock tail feather construct illustrated in Figure 4d were created using Rhino 8 software (Version 12). The spiral pyramid one‐stroke structure in Figure 5b and the desk structure shown in Figure 4d were designed using the Bio‐Architect SR software (Version 4.2).

In a typical experiment, HPC or HPC‐MA ink was drawn into a 5 mL dispensing barrel, followed by centrifugation for 5 min using a planetary mixer (AR‐100, THINKY Mixer) to avoid air bubbles. The barrel containing the HPC‐MA ink was covered with aluminum foil to protect it from light exposure throughout the process. The barrel was then mounted onto the printer, and the SC‐XG support bath was placed onto the stage. The G‐code file was imported into the printer and executed. All experiments utilized a nozzle with an inner diameter of 0.41 to 1.20 mm for printing. The printing parameters were optimized. Typically, a layer height of 0.4 mm, a line spacing of 0.3 mm, a printing speed of 2 mm/s, and an extrusion pressure of 0.2 MPa were employed, except for the experiment aimed at producing heterogeneous color intensity (Figure S20c), which used a varying printing speed of 1 ‐ 8 mm/s. After printing, the constructs were allowed to fully recover their structural color within the SC‐XG support bath for at least 10 min. To fabricate solidified cellulosic constructs, the bath containing the printed HPC‐MA construct was then placed in a UV curing chamber (365 nm, 760 W) for 200 s to ensure complete crosslinking. To mitigate the UV‐induced water evaporation effect on HPC‐MA, a UV intensity of 30% was used. Finally, the cured structures were retrieved and rinsed with 0.7 M SC solution to remove residual support material. The chainmail constructs with multiple structural colors were fabricated with multi‐material 3D printing using 64 and 66 wt.% HPC‐MA inks.

### 4.6 Fabrication of MechanoHPC Hydrogels and Actuators

To fabricate MechanoHPC hydrogels, a 55 wt.% pristine HPC solution was used as the ink, supplemented with 0.5 wt.% nigrosine that can provide broadband absorption for enhancing the contrast of the HPC structural coloration. The ink was embedded printed into a UV‐crosslinkable SC‐XG‐AAm support matrix. After printing, the container with the printed HPC and the support matrix was sealed with a transparent cover to minimize air exposure and subsequently placed in a UV curing chamber (365 nm, 760 W). The structural color of the embedded HPC can be spatially tuned by varying the HPC ink concentration from 55 to 64 wt.%, the printed HPC thickness from 1.5 mm to 3.5 mm, and the UV exposure time from 200 s to 600 s. The MechanoHPC hydrogels were UV crosslinked for 200 s (Figure 5b,c,h; Figures S24 and S25), 600 s (Figure 5d,f), and 180 s (Figure 5g).

To fabricate MechanoHPC hydrogels with DLP‐printed structural colored patterns, a layer of HPC with a thickness of 1.8 mm was first embedded printed into a SC‐XG‐AAm matrix, followed by pre‐curing in a UV curing chamber (365 nm, 760 W) for 120 s. The pre‐curing step enabled the formation of a manipulable MechanoHPC hydrogel, with the embedded HPC appearing light red at this stage. Next, the partially cured MechanoHPC hydrogel was placed in a DLP resin tank, positioned above a DLP projector (PRO4500, Wintech). Designated UV patterns, including patterns of a rotating spiral, a butterfly, *The scream*, and biomimetic snakeskin and Turing patterns were projected onto the surface of the MechanoHPC hydrogels using the DLP projector for 2 hrs. This procedure selectively induced further crosslinking in the illuminated regions. As a result, the color of the embedded HPC in the exposed areas gradually transitioned from light red to green due to the stiffer matrix and water loss, yielding a well‐defined patterned MechanoHPC hydrogel with spatially varied structural colorations. During the DLP process, the resin tank with the MechanoHPC hydrogel was hermetically sealed using a transparent film to prevent water evaporation from the hydrogel. To visualize the color‐changing effect of the MechanoHPC hydrogels when subjected to tension, the hydrogels were loaded onto a universal testing machine (C25.102, Shenzhen SANS, China) and deformed at a controlled strain rate of 500 mm/min.

To fabricate the pneumatic MechanoHPC devices, which include the information encryption device, camouflage actuator, octopus actuator, and MechanoHPC pixel display, custom models of negative molds were designed using Autodesk Inventor (Version 2025) based on the intended external geometries of these devices. Figures S29 and S31–S33 illustrate their designs and fabrications. The negative molds were 3D printed with polylactic acid using a fused deposition 3D printer (X1 Carbon, Bambu Lab). In the fabrication process, the negative mold was filled with an SC‐XG‐AAm precursor solution. Subsequently, a 55 wt.% HPC ink supplemented with 0.5 wt.% nigrosine was embedded printed into the SC‐XG‐AAm precursor matrix. For the information encryption device and the MechanoHPC octopus device, a 35 w/v% Pluronic F127 sacrificial added with 2 w/v% ascorbic acid ink was printed into the precursor matrix according to the designed geometries of hollow cavities. On the other hand, the hollow cavities of the cylindrical MechanoHPC actuator and the MechanoHPC display were created by carefully inserting plastic rod templates into the precursor matrix after printing. To solidify the SC‐XG‐AAm matrix, the entire mold with the printed inks was then crosslinked in a UV curing chamber (365 nm, 760 W) for varying durations. Specifically, a UV crosslinking time of 350 s was used for the information encryption device and the octopus device; 250 s was employed for the cylindrical camouflage actuator; 350 – 600 s was used for the MechanoHPC display. The fabricated pneumatic MechanoHPC devices were harvested from the mold. The sacrificial ink was washed away. Silicone tubes were inserted into the hollow cavities, which were then connected to a pressure source or a pneumatic controller (OB1‐C‐4400, Elveflow). Ethyl cyanoacrylate adhesive glues were applied between the silicone tubes and the hydrogels to ensure airtight sealing.

### 4.7 Control Logics of the Pneumatic MechanoHPC Hydrogel Actuators

#### 4.7.1 MechanoHPC Camouflage Actuator

The control logic of the MechanoHPC camouflage actuator is depicted in Figure S31. An RGB color sensor (TCS34725, Waveshare) was used to detect changes in the background colors and to communicate the detected RGB values to a microcontroller (Arduino UNO). When a color change was detected, a pneumatic controller (OB1 MK4, Elveflow) connected to the camouflage actuator was activated to inflate the actuator at a predefined pressure based on the detected color. This inflation process pressurized the MechanoHPC actuator, allowing it to match the background color and achieve real‐time camouflage.

#### 4.7.2 MechanoHPC Octopus Actuator

Figure S32 illustrates the design and control logic of the MechanoHPC octopus actuator. The actuator has spacers positioned beneath each arm, with electrodes placed both on and underneath each arm. Each arm contained a pneumatic channel that was connected to an air pump (JQB2438274, TCS Precision) and a solenoid valve. The electrodes were connected to a microcontroller (Arduino UNO). The spacers were used to prevent contact between the conductive arm and the electrode when the octopus actuator was not touched. When the arm was pressed, the grounded arm and the lower electrode were contacted, generating a current flow. This electrical signal was detected by the microcontroller, which then activated the air pump to inflate the arm for 200 ms, followed by a deflation period of 600 ms to complete one actuation cycle. For short press stimuli of < 1 s, only one actuation cycle was executed, while for prolonged stimuli of > 1 s, three cycles of actuation were performed.

### 4.8 Characterization

#### 4.8.1 Scanning Electron Microscopy

To observe the microstructures of the HPC‐MA samples, scanning electron microscopy was performed using a field‐emission scanning electron microscope (Merlin, Zeiss) at an accelerating voltage of 4 kV. The HPC‐MA samples were prepared by printing in one of three conditions: in a 0.7 M SC‐supplemented XG support bath, in a non‐SC‐supplemented XG bath or in air, followed by in situ crosslinking or a 10‐min waiting period before UV crosslinking for 120 s (365 nm, 760 W). After crosslinking, the samples were lyophilized and cryogenically fractured in liquid nitrogen. The cross‐sectional samples were then sputter‐coated with platinum using an ion sputtering device (Q150T ES, Quorum).

#### 4.8.2 Reflection Spectra

Diffuse reflection spectra were measured using a spectrophotometer (IdeaOptics, NOVA2S‐EX) with an integrating sphere accessory via a Y‐type bifurcated fiber‐optic assembly. All spectra were measured relative to a standard white reference.

#### 4.8.3 Mechanical Properties

The tensile mechanical properties of the SC‐XG‐AAm matrices of the MechanoHPC hydrogels were investigated using a universal testing machine (C25.102, Shenzhen SANS, China) equipped with a 500 N load cell. Rectangular specimens with a size of 50 mm (*l*) by 10 mm (*w*) by 4 mm (*t*) were prepared by casting the uncured solution into a custom mold, followed by curing in a UV chamber (365 nm, 760 W) for designated exposure durations ranging from 120 to 600 s. Uniaxial tensile tests were performed at a constant strain rate of 50 mm/min until specimen fracture. Cyclic tensile tests were carried out for 50 consecutive loading‐unloading cycles at a strain rate of 50 mm/min. All specimens were stretched to a strain of 100% in the cycle tensile tests, except for those cured for 600 s, where the maximum strain was set to 90%. The stress was calculated as the measured force divided by the initial cross‐sectional area of the sample. The strain was determined as the ratio of the change in length to the initial length.

#### 4.8.4 Rheology

Rheological measurements were performed on a stress‐controlled rheometer (DHR‐2, TA Instruments) equipped with a 40 mm parallel‐plate geometry. All measurements were conducted at a controlled temperature of 25°C. Excess solutions from the edges of the plates were trimmed to minimize edge effects, and a fixed gap of 500 µm was employed for all tests. To investigate the shear‐thinning properties of the inks and the support baths, flow sweep rotational tests were carried out over a shear rate range from 10^−2^ s^−1^ to 10^3^ s^−1^. Additionally, to examine the viscoelastic properties of the support baths, oscillatory strain sweep tests were performed over a shear strain range from 0.1% to 1000% at a constant angular frequency of 1 rad·s^−^^1^ to determine the linear viscoelastic region (LVR), the shear storage (*G′*) and loss (*G″*) moduli. The shear yield stresses (*τ_y_*) of the support baths were determined from the intersection of the tangents of the linear and nonlinear regimes of *G′*. Three‐interval thixotropy tests (3ITT) were performed under alternating low‐ and high‐strain conditions. A low oscillatory strain (amplitude strain = 1%) was applied for 60 s, followed by a high oscillatory strain (amplitude strain = 500%) for 60 s. This alternating application of strains was repeated for five consecutive cycles at a frequency of 1 Hz. All rheological data were collected and processed using TRIOS software (TRIOS 5.1.1, TA Instruments), and each measurement was repeated at least three times.

#### 4.8.5 Water Activity

Water activity was determined using an AquaLab 4TE analyzer (METER Group, Inc., USA) at 25°C. The measurements were based on the chilled‐mirror dew point method, and each sample was analyzed in triplicate.

#### 4.8.6 Optical Microscopy and Photography

Optical photographs and videos were taken by a digital camera (Alpha 7IV, SONY) under white light. Microscopy images of HPC‐MA filaments fabricated via in‐air printing and ATPS embedded printing were acquired using a toolmakers microscope (GP‐200C, Gaopin).

## Conflicts of Interest

The authors declare no conflicts of interest.

## Supporting information




**Supporting File 1**: adma72413‐sup‐0001‐SuppMat.pdf.

**Supporting File 2**: adma72413‐sup‐0002‐Movie S1.mp4.

**Supporting File 3**: adma72413‐sup‐0003‐Movie S2.mp4.

**Supporting File 4**: adma72413‐sup‐0004‐Movie S3.mp4.

**Supporting File 5**: adma72413‐sup‐0005‐Movie S4.mp4.

**Supporting File 6**: adma72413‐sup‐0006‐Movie S5.mp4.

**Supporting File 7**: adma72413‐sup‐0007‐Movie S6.mp4.

**Supporting File 8**: adma72413‐sup‐0008‐Movie S7.mp4.

**Supporting File 9**: adma72413‐sup‐0009‐Movie S8.mp4.

**Supporting File 10**: adma72413‐sup‐0010‐Movie S9.mp4.

## Data Availability

The data that support the findings of this study are available from the corresponding author upon reasonable request.

## References

[1] S. Kinoshita , S. Yoshioka , and J. Miyazaki , “Physics of Structural Colors,” Reports on Progress in Physics 71 (2008): 076401.

[2] A. G. Dumanli and T. Savin , “Recent Advances in the Biomimicry of Structural Colours,” Chemical Society Reviews 45 (2016): 6698–6724, 10.1039/C6CS00129G. 27510041

[3] S. H. Choi , D. Kim , and Y. Lee , “Bioinspired Dynamic Colour Change,” Nature Reviews Bioengineering 3 (2025): 579–595.

[4] G. Bogdanov , A. A. Strzelecka , and N. Kaimal , “Gradient Refractive Indices Enable Squid Structural Color and Inspire Multispectral Materials,” Science 388 (2025): 1389–1395, 10.1126/science.adn1570. 40570125

[5] X. Y. Zhang , K. M. Zhou , Z. J. Zhao , and Y. J. Lin , “Printable Photonic Materials and Devices for Smart Healthcare,” Advanced Materials 37 (2025): 2418729, 10.1002/adma.202418729. 40045686

[6] C. A. Williams , R. M. Parker , A. Kyriacou , M. Murace , and S. Vignolini , “Inkjet Printed Photonic Cellulose Nanocrystal Patterns,” Advanced Materials 36 (2024): 2307563. 10.1002/adma.202307563 37965844

[7] K. Li , T. Li , and T. Zhang , “Facile Full‐Color Printing with a Single Transparent Ink,” Science Advances 7 (2021): abh1992. 10.1126/sciadv.abh1992 PMC8457659 34550746

[8] A. F. Demirörs , E. Poloni , and M. Chiesa , “Three‐Dimensional Printing of Photonic Colloidal Glasses into Objects with Isotropic Structural Color,” Nature Communications 13 (2022): 4397. 10.1038/s41467-022-32060-2 PMC9338281 35906208

[9] Z. H. Zhang , C. Wang , Q. Wang , Y. J. Zhao , and L. R. Shang , “Cholesteric cellulose liquid crystal ink for three‐dimensional structural coloration,” Proccedings of the National Academy of the United States of America 119 23 (2022) e2204113119. 10.1073/pnas.2204113119 PMC9191658 35639690

[10] K. George , M. Esmaeili , J. Y. Wang , N. Taheri‐Qazvini , A. Abbaspourrad , and M. Sadati , “3D printing of responsive chiral photonic nanostructures” Proceedings of the National Academy of Sciences of the United States of America 120 12 (2023) e2220032120. 36917662 10.1073/pnas.2220032120 PMC10041133

[11] J. B. Kim , H. Y. Lee , C. Chae , S. Y. Lee , and S. H. Kim , “Advanced Additive Manufacturing of Structurally‐Colored Architectures,” Advanced Materials 36 (2024): 2307917, 10.1002/adma.202307917. 37909823

[12] J. B. Kim , C. Chae , S. H. Han , S. Y. Lee , and S. H. Kim , “Direct Writing of Customized Structural‐Color Graphics with Colloidal Photonic Inks,” Science Advances 7 (2021): abj8780, 10.1126/sciadv.abj8780. PMC8612532 34818030

[13] J. Zhang , Y. P. Qin , and Y. T. Ou , “Injectable Granular Hydrogels as Colloidal Assembly Microreactors for Customized Structural Colored Objects” Angewandte Chemie, International Edition 61 34 (2022) e202206339. 35735050 10.1002/anie.202206339

[14] B. B. Patel , D. J. Walsh , and D. Kim , “Tunable Structural Color of Bottlebrush Block Copolymers through Direct‐Write 3D Printing from Solution,” Science Advances 6 (2020): aaz7202. 10.1126/sciadv.aaz7202 PMC7286684 32577511

[15] Y. Zhang , L. Zhang , and C. Zhang , “Continuous Resin Refilling and Hydrogen Bond Synergistically Assisted 3D Structural Color Printing,” Nature Communications 13 (2022): 7095, 10.1038/s41467-022-34866-6. PMC9675848 36402778

[16] J. H. Yi , S. Q. Yang , L. Yue , and I. M. Lei , “Digital light processing 3D printing of flexible devices: actuators, sensors and energy devices” Microsystems & Nanoengineering 11 1 (2025) 51. 40108126 10.1038/s41378-025-00885-8 PMC11923083

[17] Y. Liu , H. Wang , and J. Ho , “Structural Color Three‐Dimensional Printing by Shrinking Photonic Crystals,” Nature Communications 10 (2019): 4340, 10.1038/s41467-019-12360-w. PMC6761189 31554803

[18] R. M. Parker , T. G. Parton , C. L. C. Chan , M. M. Bay , B. Frka‐Petesic , and S. Vignolini , “Bioinspired Photonic Materials from Cellulose: Fabrication, Optical Analysis, and Applications,” Accounts of Materials Research 4 (2023): 522–535, 10.1021/accountsmr.3c00019. 37383657 PMC10294254

[19] B. Frka‐Petesic , T. G. Parton , and C. Honorato‐Rios , “Structural Color from Cellulose Nanocrystals or Chitin Nanocrystals: Self‐Assembly, Optics, and Applications,” Chemical Reviews 123 (2023): 12595–12756, 10.1021/acs.chemrev.2c00836. 38011110 PMC10729353

[20] R. S. Werbowyj and D. G. Gray , “Ordered Phase Formation in Concentrated Hydroxpropylcellulose Solutions,” Macromolecules 13 (1980): 69–73, 10.1021/ma60073a014.

[21] C. L. C. Chan , I. M. Lei , and G. T. van de Kerkhof , “3D Printing of Liquid Crystalline Hydroxypropyl Cellulose—Toward Tunable and Sustainable Volumetric Photonic Structures,” Advanced Functional Materials 32 (2022): 2108566, 10.1002/adfm.202108566.

[22] Z. H. Zhang , Z. Y. Chen , Y. Wang , and Y. J. Zhao , “Bioinspired Conductive Cellulose Liquid‐Crystal Hydrogels as Multifunctional Electrical Skins,” Proceedings of the National Academy of Sciences 117 (2020): 18310–18316, 10.1073/pnas.2007032117. PMC7414159 32675247

[23] H. Yi , S. H. Lee , and H. Ko , “Ultra‐Adaptable and Wearable Photonic Skin Based on a Shape‐Memory, Responsive Cellulose Derivative,” Advanced Functional Materials 29 (2019): 1902720, 10.1002/adfm.201902720.

[24] Z. H. Zhang , Z. Y. Chen , Y. Wang , Y. J. Zhao , and L. R. Shang , “Cholesteric Cellulose Liquid Crystals with Multifunctional Structural Colors” Advanced Functional Materials 32 12 (2022) 2107242.

[25] C. H. Barty‐King , M. Burgonse , S. Vignolini , J. Baumberg , and M. De Volder , “Mechanochromic, Low‐Cost, and Structurally Colored Displays Using Biodegradable Hydroxypropyl Cellulose,” Advanced Materials 37 (2025): 2418880, 10.1002/adma.202418880. 40346773 PMC12288781

[26] H. L. Liang , M. M. Bay , and R. Vadrucci , “Roll‐to‐Roll Fabrication of Touch‐Responsive Cellulose Photonic Laminates,” Nature Communications 9, no. 1 (2018): 4632, 10.1038/s41467-018-07048-6. PMC6219516 30401803

[27] Y. Huang , Y. Qian , and Y. Chang , “Intense Left‐Handed Circularly Polarized Luminescence in Chiral Nematic Hydroxypropyl Cellulose Composite Films,” Advanced Materials 36 (2024): 2308742. 10.1002/adma.202308742 38270293

[28] S. Y. Ming , X. T. Zhang , and C. L. C. Chan , “Exploiting the Thermotropic Behavior of Hydroxypropyl Cellulose to Produce Edible Photonic Pigments” Advanced Sustainable Systems 7 4 (2023) 2200469.

[29] S. G. Fine , S. E. Branovsky , and C. A. C. Chazot , “Structural Color out of the Blue: a Quantitative Framework for the Self‐Assembly Kinetics of Cholesteric Cellulosic Mesophases,” Biomacromolecules 25 (2024): 4977–4990, 10.1021/acs.biomac.4c00411. 38949966

[30] Q. Wang , C. Wang , and Z. L. Fang , “Hydroxypropyl Cellulose Assembled Microspheres as Structural Color Barcodes from Revolving Microfluidics,” Advanced Science 12 32 (2025) e06556. 40464340 10.1002/advs.202506556 PMC12407317

[31] Q. Wang , Z. H. Zhang , C. Wang , X. Y. Yang , Z. L. Fang , and L. R. Shang , “Bioinspired Confined Assembly of Cellulosic Cholesteric Liquid Crystal Bubbles” Advanced Science 11 11 (2024) 2308442. 38225706 10.1002/advs.202308442 PMC10953211

[32] X. Ma , B. Wu , L. Hou , and P. Wu , “Edible Structurally Colored Plastics,” Acs Nano 26 (2025): 23945–23954. 10.1021/acsnano.5c05346 40561459

[33] X. Deng , C. Qi , and S. Meng , “All‐Aqueous Embedded 3D Printing for Freeform Fabrication of Biomimetic 3D Constructs,” Advanced Materials 36 (2024): 2406825. 10.1002/adma.202406825 39520386

[34] Y. Fu , Z. Li , S. Zhao , H. Hou , and Y. Chai , “Reconfigurable Aqueous 3D Printing with Adaptive Dual Locks,” Science Advances 10 (2024): adk4080, 10.1126/sciadv.adk4080. PMC11042732 38657077

[35] S. Zhang , C. Qi , and W. Zhang , “In Situ Endothelialization of Free‐Form 3D Network of Interconnected Tubular Channels via Interfacial Coacervation by Aqueous‐in‐Aqueous Embedded Bioprinting,” Advanced Materials 35 (2023): 2209263, 10.1002/adma.202209263. 36448877

[36] G. Luo , Y. Yu , Y. Yuan , X. Chen , Z. Liu , and T. Kong , “Freeform, Reconfigurable Embedded Printing of all‐Aqueous 3D Architectures,” Advanced Materials 31 (2019): 1904631, 10.1002/adma.201904631. 31609497

[37] G. Tang , Z. Luo , and L. Lian , “Liquid‐Embedded (bio) Printing of Alginate‐Free, Standalone, Ultrafine, and Ultrathin‐Walled Cannular Structures,” Proceedings of the National Academy of Sciences 120 (2023): 2206762120. 10.1073/pnas.2206762120 PMC9963289 36745792

[38] M. Iqbal , Y. F. Tao , and S. Y. Xie , “Aqueous Two‐Phase System (ATPS): an Overview and Advances in Its Applications,” Biological Procedures Online 18 (2016): 18, 10.1186/s12575-016-0048-8. 27807400 PMC5084470

[39] A. Glyk , T. Scheper , and S. Beutel , “Influence of Different Phase‐Forming Parameters on the Phase Diagram of Several PEG–Salt Aqueous Two‐Phase Systems,” Journal of Chemical & Engineering Data 59 (2014): 850–859, 10.1021/je401002w.

[40] Q. Qin and Y. Xu , “Hydroxypropyl Cellulose‐Based Meter‐Long Structurally Colored Fibers for Advanced FabricsHydroxypropyl Cellulose‐Based Meter‐Long Structurally Colored Fibers for Advanced Fabrics” Advanced Science 11 46 (2024) 2404761. 39432405 10.1002/advs.202404761 PMC11633506

[41] Z. Zhang , Q. Wang , Y. Li , C. Wang , X. Yang , and L. Shang , “Cholesteric Cellulose Liquid Crystal Fibers by Direct Drawing,” Research 7 (2024): 0527. 39512448 10.34133/research.0527 PMC11541814

[42] I. M. Lei , D. Zhang , W. X. Gu , J. Liu , Y. L. Zi , and Y. Y. S. Huang , “Soft Hydrogel Shapeability via Supportive Bath Matching in Embedded 3D Printing” Advanced Materials Technologies 8 15 (2023) 2300001.

[43] M. Wehner , R. L. Truby , and D. J. Fitzgerald , “An Integrated Design and Fabrication Strategy for Entirely Soft, Autonomous Robots,” Nature 536 (2016): 451–455, 10.1038/nature19100. 27558065

[44] Y. Hui , Y. Yao , and Q. Qian , “Three‐Dimensional Printing of Soft Hydrogel Electronics,” Nature Electronics 5 (2022): 893–903, 10.1038/s41928-022-00887-8.

[45] X. J. Xie , Z. G. Xu , X. Yu , H. Jiang , H. J. Li , and W. Q. Feng , “Liquid‐in‐liquid printing of 3D and mechanically tunable conductive hydrogels” Nature Communications 14 1 (2023) 4289. 10.1038/s41467-023-40004-7 PMC10354067 37463898

[46] A. Lee , A. R. Hudson , and D. J. Shiwarski , “3D bioprinting of Collagen to Rebuild Components of the human Heart,” Science 365 (2019): 482–487, 10.1126/science.aav9051. 31371612

[47] D. J. Shiwarski , A. R. Hudson , and J. W. Tashman , “3D bioprinting of Collagen‐Based High‐Resolution Internally Perfusable Scaffolds for Engineering Fully Biologic Tissue Systems,” Science Advances 11 (2025): adu5905. 10.1126/sciadv.adu5905 PMC12017336 40267204

[48] A. K. Grosskopf , R. L. Truby , H. Kim , A. Perazzo , J. A. Lewis , and H. A. Stone , “Viscoplastic Matrix Materials for Embedded 3D Printing,” ACS Applied Materials & Interfaces 10 (2018): 23353–23361, 10.1021/acsami.7b19818. 29493215

[49] T. Balcerowski , B. Ozbek , O. Akbulut , and A. G. Dumanli , “Hierarchical Organization of Structurally Colored Cholesteric Phases of Cellulose via 3D Printing,” Small 19 (2023): 2205506. 10.1002/smll.202205506 36504424

[50] H. N. Ren , I. O. Sodipo , and A. G. Dumanli , “Stretchable Cellulosic Cholesteric Liquid Crystal Filaments with Color Response,” ACS Applied Polymer Materials 7 (2025): 4093–4098, 10.1021/acsapm.4c02719. 40242049 PMC11997954

[51] L. D. C. de Castro , J. Lub , O. N. Oliveira , and A. P. H. J. Schenning , “Mechanochromic Displays Based on Photoswitchable Cholesteric Liquid Crystal Elastomers” Angewandte Chemie, International Edition 64 1 (2025) e202413559. 39188146 10.1002/anie.202413559 PMC11701355

[52] Y. Geng , R. Kizhakidathazhath , and J. P. F. Lagerwall , “Robust Cholesteric Liquid Crystal Elastomer Fibres for Mechanochromic Textiles,” Nature Materials 21 (2022): 1441–1447, 10.1038/s41563-022-01355-6. 36175519 PMC9712110

[53] A. Ng , R. Telles , and K. S. Riley , “Coaxial Direct Ink Writing of Cholesteric Liquid Crystal Elastomers in 3D Architectures,” Advanced Materials 37 (2025): 2416621. 39865794 10.1002/adma.202416621 PMC11899511

[54] B. H. Miller , H. L. Liu , and M. Kolle , “Scalable Optical Manufacture of Dynamic Structural Colour in Stretchable Materials,” Nature Materials 21 (2022): 1014–1018, 10.1038/s41563-022-01318-x. 35915162

[55] D. A. Davis , A. Hamilton , and J. L. Yang , “Force‐Induced Activation of Covalent Bonds in Mechanoresponsive Polymeric Materials,” Nature 459 (2009): 68–72, 10.1038/nature07970. 19424152

[56] V. C. Ritter , S. M. McDonald , A. V. Dobrynin , S. L. Craig , and M. L. Becker , “Mechanochromism and Strain‐Induced Crystallization in Thiol‐Yne‐Derived Stereoelastomers,” Advanced Materials 35 (2023): 2302163. 10.1002/adma.202302163 37399511

[57] C. H. Barty‐King , C. L. C. Chan , and R. M. Parker , “Mechanochromic, Structurally Colored, and Edible Hydrogels Prepared from Hydroxypropyl Cellulose and Gelatin,” Advanced Materials 33 (2021): 2102112. 34323315 10.1002/adma.202102112 PMC11468689

[58] J. Liu , W. Li , Y. She , S. Blanchard , and S. Lin , “Fatigue‐Resistant Mechanoresponsive Color‐Changing Hydrogels for Vision‐Based Tactile Robots,” Advanced Materials 37, no. 49 (2024): 2407925, 10.1002/adma.202407925. 39328076 PMC12691901

