# Supplementary material for: Freeform Manufacturing of Plant‐Based Structural Colors for Scalable Photonic and Mechanochromic Devices
Source: Adv Mater. 2026 Feb 6;38(35):e19692. doi: 10.1002/adma.202519692 (PMC13288203; doi:10.1002/adma.202519692)
Supplement: Supplementary file 1 — Supporting File 1: adma72413‐sup‐0001‐SuppMat.pdf. [file ADMA-38-e19692-s009.pdf]

Freeform Manufacturing of Plant-Based Structural Colors for Scalable Photonic and Mechanochromic Devices

Supplementary information

Xiao Song[1,2], Peiqi Niu[1,2], Wenxi Gu[1,2], Chun Lam Clement Chan[3,4], Jiuhong Yi[1,2], Xu Liu[1,2], Peng Tan[1,2], Chon In Haydn Cheong[5], Qingwen Guan[1,2], Dan Fang[6], Bingpu Zhou[6], Zi Liang Wu[7], Ji Liu[8], Yan Yan Shery Huang[5]* and Iek Man Lei[1,2]*

[1]Department of Electromechanical Engineering, University of Macau, Macau 999078, China.

[2]Centre for Artificial Intelligence and Robotics, University of Macau, Macau 999078, China.

[3]Stratingh Institute for Chemistry, University of Groningen, 9700 AB, Groningen, Netherlands.

[4]Department of Chemical and Biological Engineering, Princeton University, Princeton, NJ 08544, USA.

[5]Department of Engineering, University of Cambridge, Cambridge, CB2 1PZ, UK.

[6]Joint Key Laboratory of the Ministry of Education, Institute of Applied Physics and Materials Engineering, University of Macau, Macau, 999078 China.

[7]Ministry of Education Key Laboratory of Macromolecular Synthesis and Functionalization, Department of Polymer Science and Engineering, Zhejiang University, Hangzhou 310058, China.

[8]Department of Mechanical and Energy Engineering, Southern University of Science and Technology, Shenzhen, 518055 China.

Corresponding authors: ieklei@um.edu.mo, yysh2@cam.ac.uk.

Table S1| Comparison of the fabrication techniques for plant-based structural colored materials, including cellulose nanocrystals (CNC) and hydroxypropyl cellulose (HPC), and the ATPS embedded printing technique developed in this work. Reprinted with permission from refs[1-11].

Ref	Materials	Fabrication techniques	Attainable structures and features	Examples
Droguet et al., *Nat. Mater.*, **2022**, 21(3), 352-358.[1]	CNC	Roll-to-roll coating	• Films	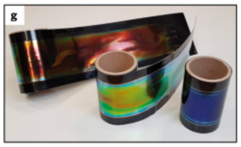
Parker et al., *Nat. Commun.*, **2022**, 13(1), 3378.[2]	CNC	Microfluidics	• Microparticles	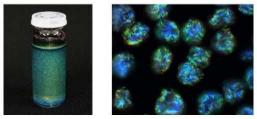
Song et al., *Adv. Mater.*, **2025**, 37(22), 2416607.[3]	CNC	Spraying	• Microdroplets	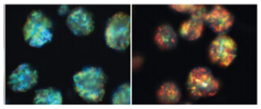
Williams et al., *Adv. Mater.*, **2024**, 36(1), 2307563.[4]	CNC	Inkjet printing	• 2D patterns	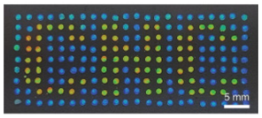
Liang et al., *Nat. Comm.* **2018**, 9(1), 4632.[5]	HPC	Coating	• Films	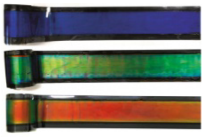
Wang et al., *Adv. Sci.*, **2025**, 12, e06556.[6]	HPC	Microfluidics	• Droplets	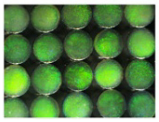
Wang et al., *Adv Sci.*, **2024**, 11, 2308442.[7]	HPC		• Microcapsules	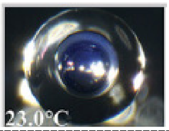
Ma et al., *ACS Nano,* **2025**, 19(26), 23945.[8]	HPC	Injection molding	• 3D structures with non-suspended features; • Limited customizability; • Limited multicolor designability.	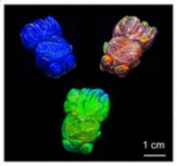
George et al., *PNAS*, **2023**, 120(12), e 2220032120.[9]	HPC	In-air direct ink writing	• Simple 2D structures	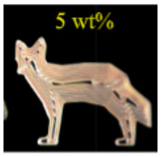

Chan et al., *Adv. Funct. Mater.*, **2022**, 32(15), 2108566.[10]	HPC		• Simple 3D structures with non-suspended features;<br>• Color loss issues over time;<br>• Restricted processing time;<br>• Limited potential for producing large-scale constructs.	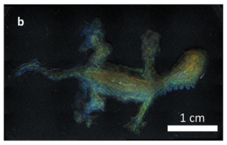
Zhang et al., *PNAS*, **2022**, 119(23), e2204113119.[11]	HPC		• Simple 3D structures with non-suspended features	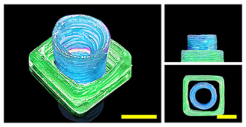
**This work**	HPC	ATPS embedded printing	✓ Intricate 3D structures with suspended features;<br>✓ Good resolution of 48 µm;<br>✓ Good color fidelity;<br>✓ Enable prolonged printing time for large-scale and complex constructs;<br>✓ Enable post-extrusion recovery of cholesteric domains;<br>✓ Multi-color designability;<br>✓ Enable integration with multiple functional inks for fabricating devices with unique functionalities (e.g., actuation capabilities).	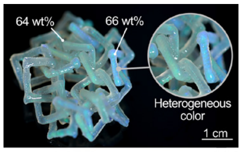 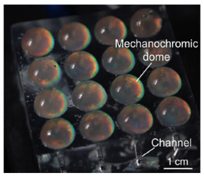

Table S2| Equations used for generating the print path of one-stroke constructs.

Curves	Equations and parameters
**Spherical helix**	$$x(t) = \sqrt{r^2 - (\frac{rt}{2\pi s})^2} \times \cos(t)$$ $$y(t) = \sqrt{r^2 - (\frac{rt}{2\pi s})^2} \times \sin(t)$$ $$z(t) = \frac{rt}{2\pi s}$$ where $t \in [-2\pi s, 2\pi s]$, the radius of the sphere *r* = 8 and the spiral density control parameter *s* = 3.
**Heart-shaped spiral**	The heart-shaped spiral contains three segments (*i* = 1, 2, 3), each described by the following equations. $$x(t) = k_i \times 16sin^3(t)$$ $$y(t) = k_i \times [13\,cos(t) - 5\,cos(2t) - 2\,cos(3t) - cos(4t)]$$ $$z(t) = \frac{t}{2}$$ where $t \in [2\pi(i-1), 2\pi i]$ for $i = 1, 2, 3$, and the scaling factors k_i are defined as: $$k_1 = \frac{1}{4}, k_2 = \frac{1}{3}, k_3 = \frac{1}{2}$$
**Rectangular spiral**	The rectangular spiral consists of 5 coils, starting at the initial point $(x_0, y_0, z_0) = (0, 0, 0)$. Each side of the spiral increases in length by 1 mm and the z-coordinate is incremented by 1 mm at each corner.
**Butterfly curve**	$$x(t) = 10 \times \cos\,(2t) \times cos(t)$$ $$y(t) = 10 \times \cos(\pi/4) \times cos(2t) \times sin(t)$$ $$z(t) = 10 \times \sin(\pi/4) \times cos(2t) \times sin(t)$$ where $t \in \left[-\frac{\pi}{4}, \frac{7\pi}{4}\right]$.

Table S3| Density of cholesteric HPC solutions and kosmotropic salt solutions at the optimal concentrations that can form ATPS with cholesteric HPC.

Materials	Density (g/ml)
61 wt% HPC	1.154 ± 0.002
64 wt% HPC	1.159 ± 0.001
66 wt% HPC	1.169 ± 0.003
0.7 M Sodium citrate	1.126 ± 0.005
1.0 M Sodium sulfate	1.108 ± 0.002
1.0 M Disodium phosphate	1.113 ± 0.005
0.8 M Potassium citrate	1.118 ± 0.001
1.8 M Magnesium sulfate	1.207 ± 0.004

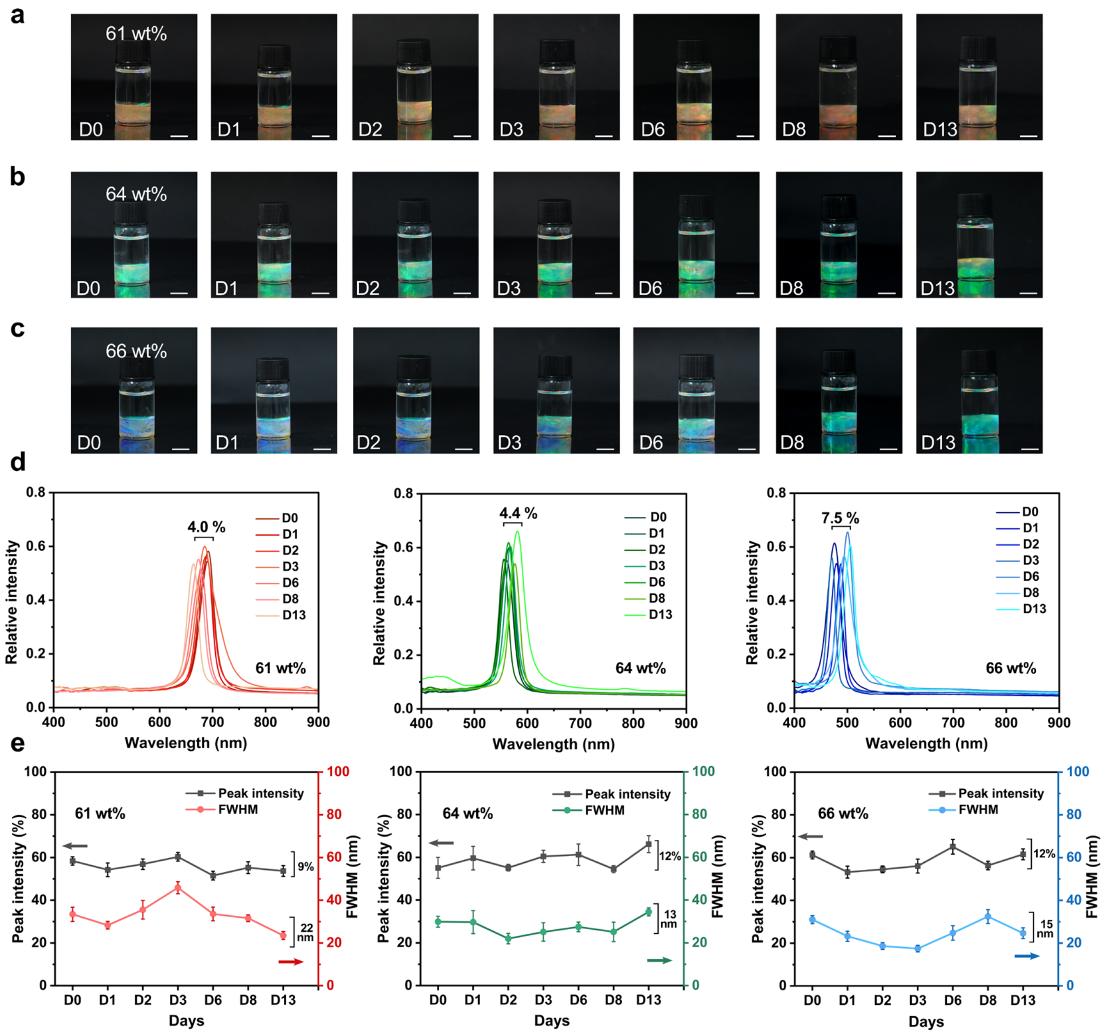

Figure S1| Stability of the salt/HPC ATPS over 13 days. a-c) Photographs illustrating the stability of the salt/HPC ATPSs and the well-preserved structural color of the HPC-rich phases over 13 days (D0, day 0 – D13, day 13). The salt phase consisted of 0.7 M sodium citrate, while the polymer-rich phase was composed of a) 61 wt%, b) 64 wt%, and c) 66 wt% HPC. Scale bars, 5 mm. **d)** Corresponding reflection spectra of the HPC-rich phases, indicating a less than 7.5% change in the peak wavelength for all HPC concentrations over 13 days. **e)** Changes in the peak intensity and full width at half maximum (FWHM) of the reflection spectra over 13 days.

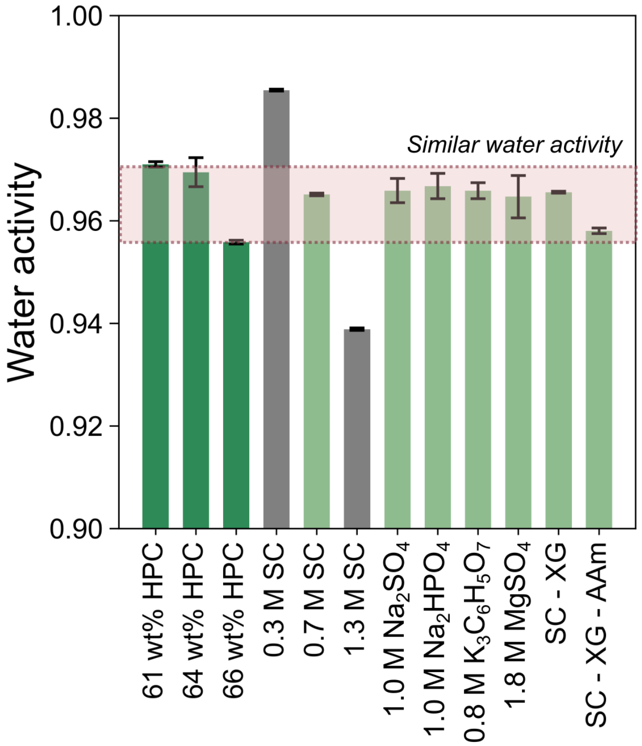

Figure S2| Water activity measurements of cholesteric HPC solutions and kosmotropic salt solutions. The cholesteric HPC solutions have similar water activity to the kosmotropic salt solutions at optimal concentrations, which can form ATPS with the HPC. SC-XG and SC-XG-AAm refer to 0.7 M SC-1.5 w/v% XG and 0.7 M SC-1.5 w/v% XG-10 w/v% AAm, which were used for ATPS embedded printing freeform photonic constructs and mechanochromic devices in this work.

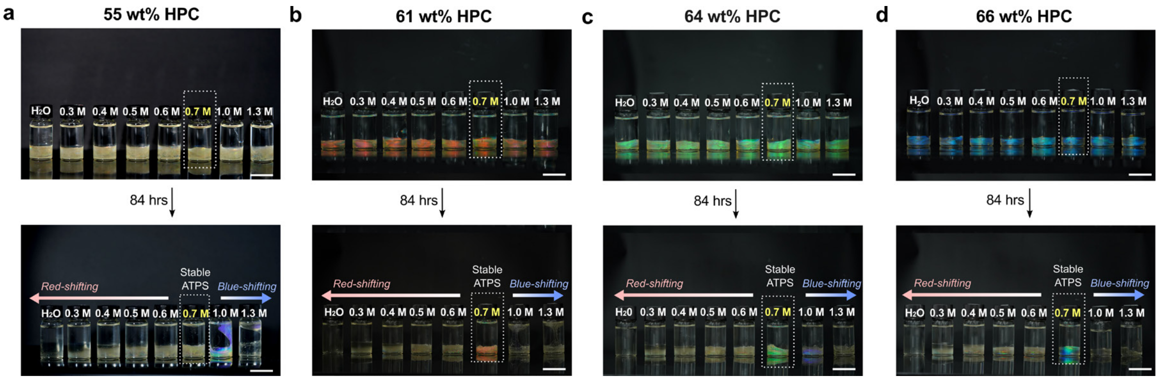

Figure S3| Influence of sodium citrate concentration on the stability of ATPSs. ATPSs containing **a)** 55 wt%, **b)** 61 wt%, **c)** 64 wt% and **d)** 66 wt% HPC as the polymer-rich phases. The concentrations of sodium citrate used are indicated in the photographs. A salt-rich phase of 0.7 M sodium citrate solution resulted in a stable ATPS, with no structural color change observed over 84 hrs. In contrast, when the sodium citrate concentration was inappropriate, a shift in the structural color of HPC occurred. A blue-shifting effect was observed when the salt-rich phase had a high SC concentration (i.e., > 1.0 M). This effect was similarly noted when SC was added directly to the HPC (**Figure S4**). The 55 wt% and 61 wt% HPC phases float in 1 M and 1.3 M SC solutions due to higher densities of the SC solutions. Scale bars, 10 mm.

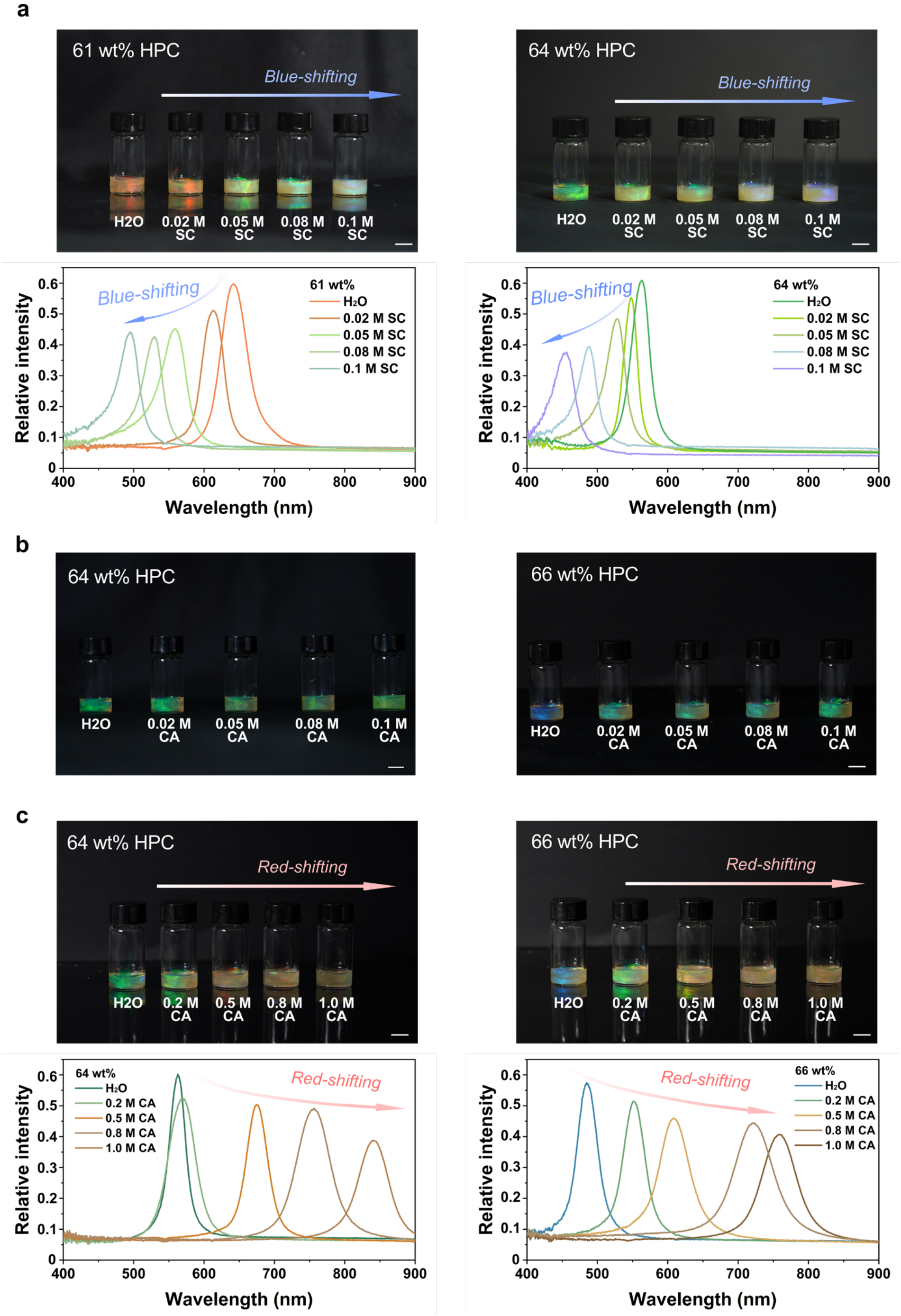

Figure S4| Kosmotropic effect of sodium citrate. a) Blue shifting effect and reflection spectra when increasing the SC concentration from 0 M to 0.1 M in 61 wt% and 64 wt% HPC. Further increases in SC concentration led to poor HPC solubility. Notably, the blue-shifting effect contrasts with that of citric acid, which induces a red-shifting effect. This effect is attributed to the high affinity of sodium citrate for water, which enhances the hydrophobic interactions between HPC polymer chains and consequently reduces the cholesteric pitch. **b)** Slight red shifting effect when increasing the citric acid (CA) concentration from 0 M to 0.1 M. **c)** Strong red shifting effect and reflection spectra when increasing the CA concentration from 0 M to 1 M. Scale bars, 5 mm.

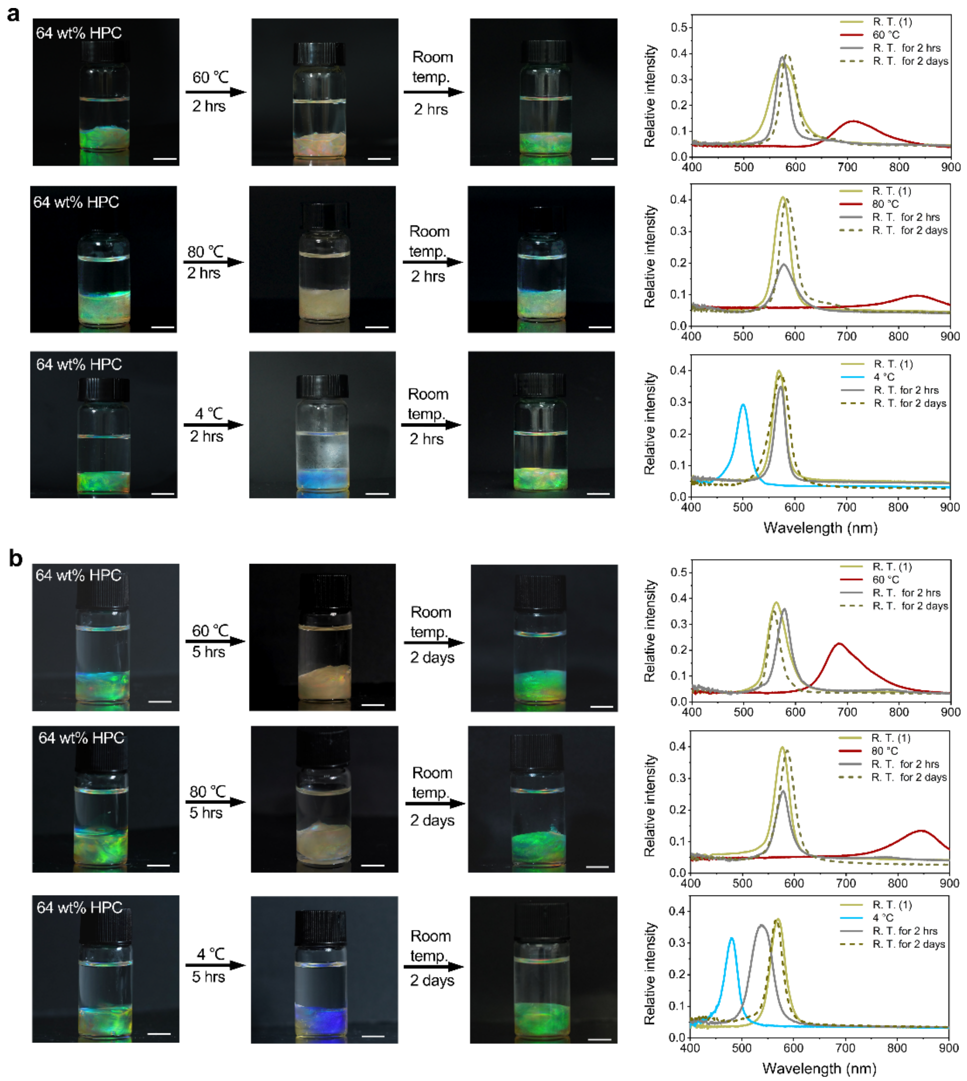

Figure S5| Recoverability of the ATPS after thermal treatment. Photographs depicting the appearances of the ATPSs before and after different thermal treatments, and their corresponding reflection spectra. **a)** Thermal treatment for 2 hrs. **b)** Thermal treatment for 5 hrs. Scale bars, 5 mm. Curves labelled "R.T. (1)" in the reflection spectra represent measurements taken before thermal treatment at room temperature, while "R.T. for 2 hrs" and "R.T. for 2 days" correspond to measurements taken after recovery for 2 hrs and 2 days from thermal treatment, respectively.

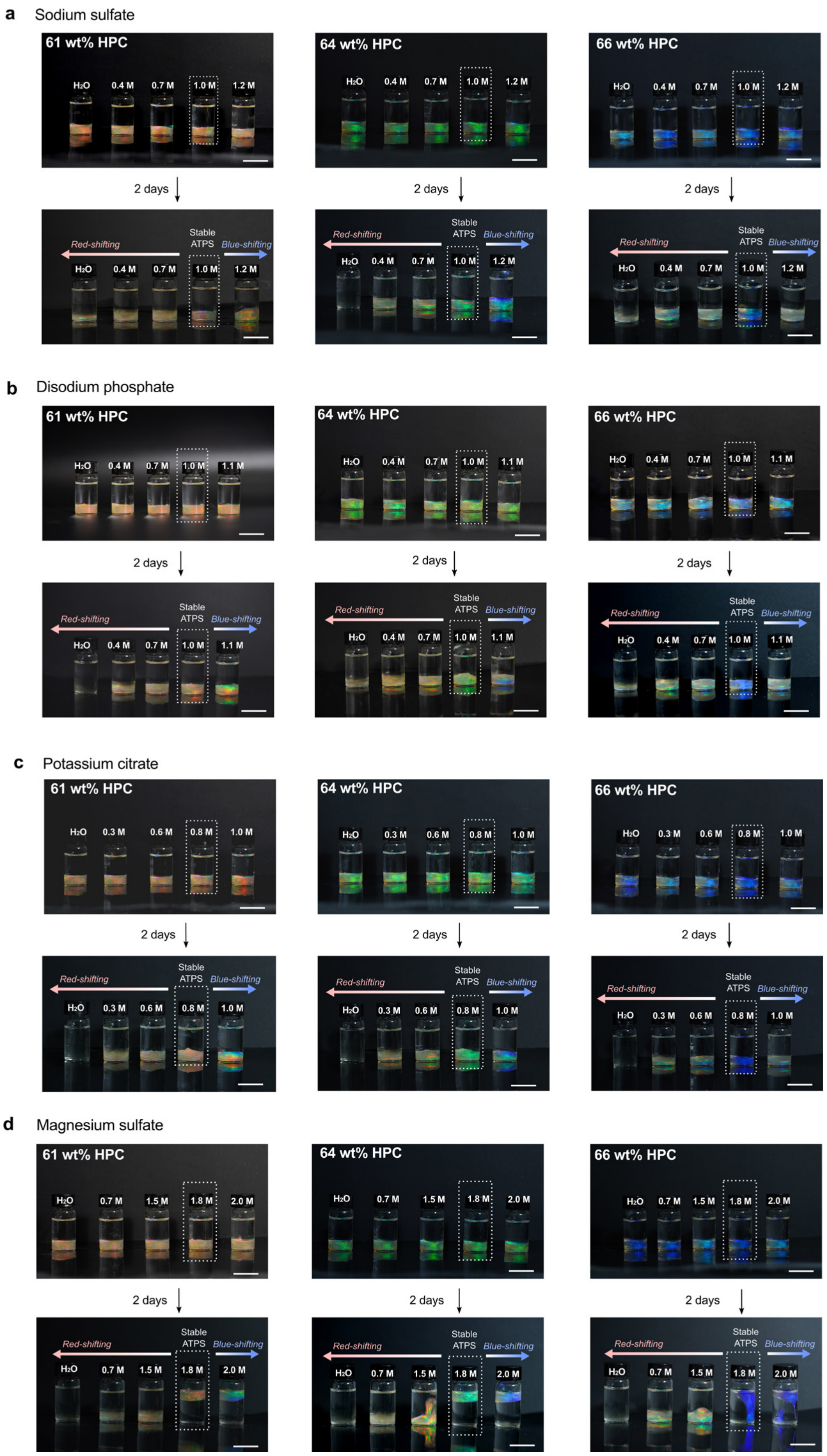

Figure S6| ATPS formation with various kosmotropic salts. a) Sodium sulfate. **b)** Disodium phosphate. **c)** Potassium citrate. **d)** Magnesium sulfate**.** The finding shows that 1.0 M sodium sulfate, 1.0 M disodium phosphate, 0.8 M potassium citrate and 1.8 M magnesium sulfate solutions can form ATPSs with 61 – 66 wt% HPC. The HPC phases floated in the 1.8 M magnesium sulfate solutions due to the higher density of the salt solutions compared to the HPC phases, as indicated in the density measurements (**Table S3**). Scale bars, 10 mm.

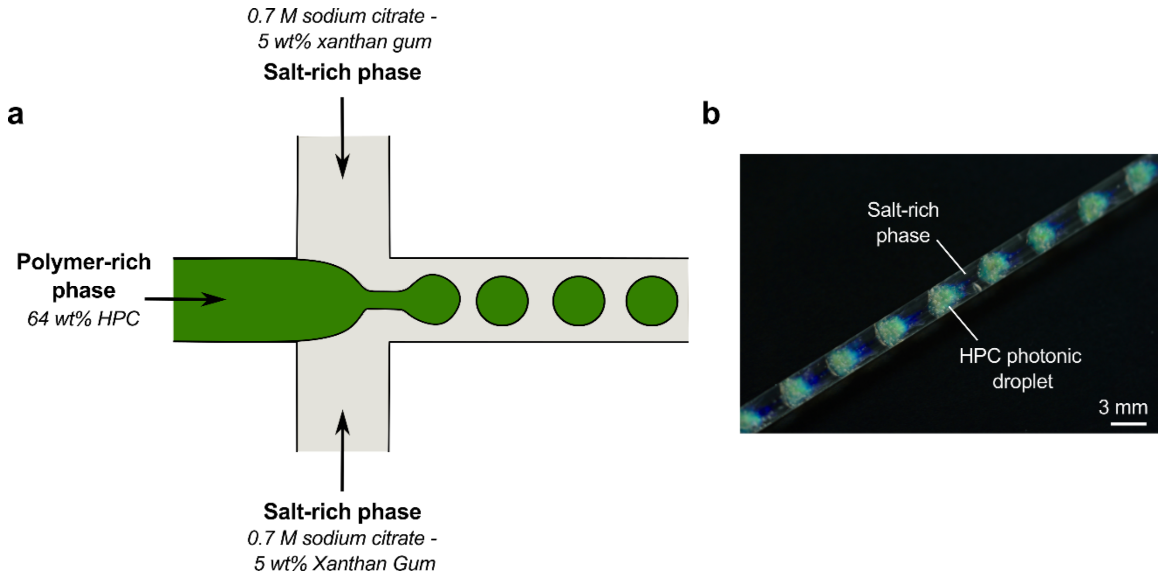

Figure S7| Fabrication of photonic HPC droplets using ATPS. a) Schematic illustrating the experimental setup. The salt-rich phase was composed of 0.7 M sodium citrate and 5 wt% xanthan gum as a thickener. The polymer-rich phase consisted of 64 wt% HPC. **b)** Photographs of the photonic HPC droplets.

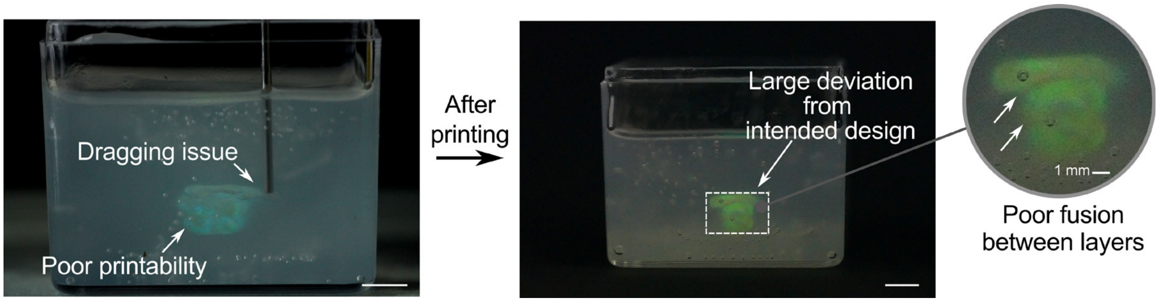

Figure S8| Issues of using oil-based support baths for 3D printing HPC photonic structures. The high interfacial tension between the hydrophobic bath and hydrophilic HPC ink resulted in poor filament fusion and unsatisfactory printability. The printed structures had significant dimensional deviations from the intended design. Scale bars, 5 mm.

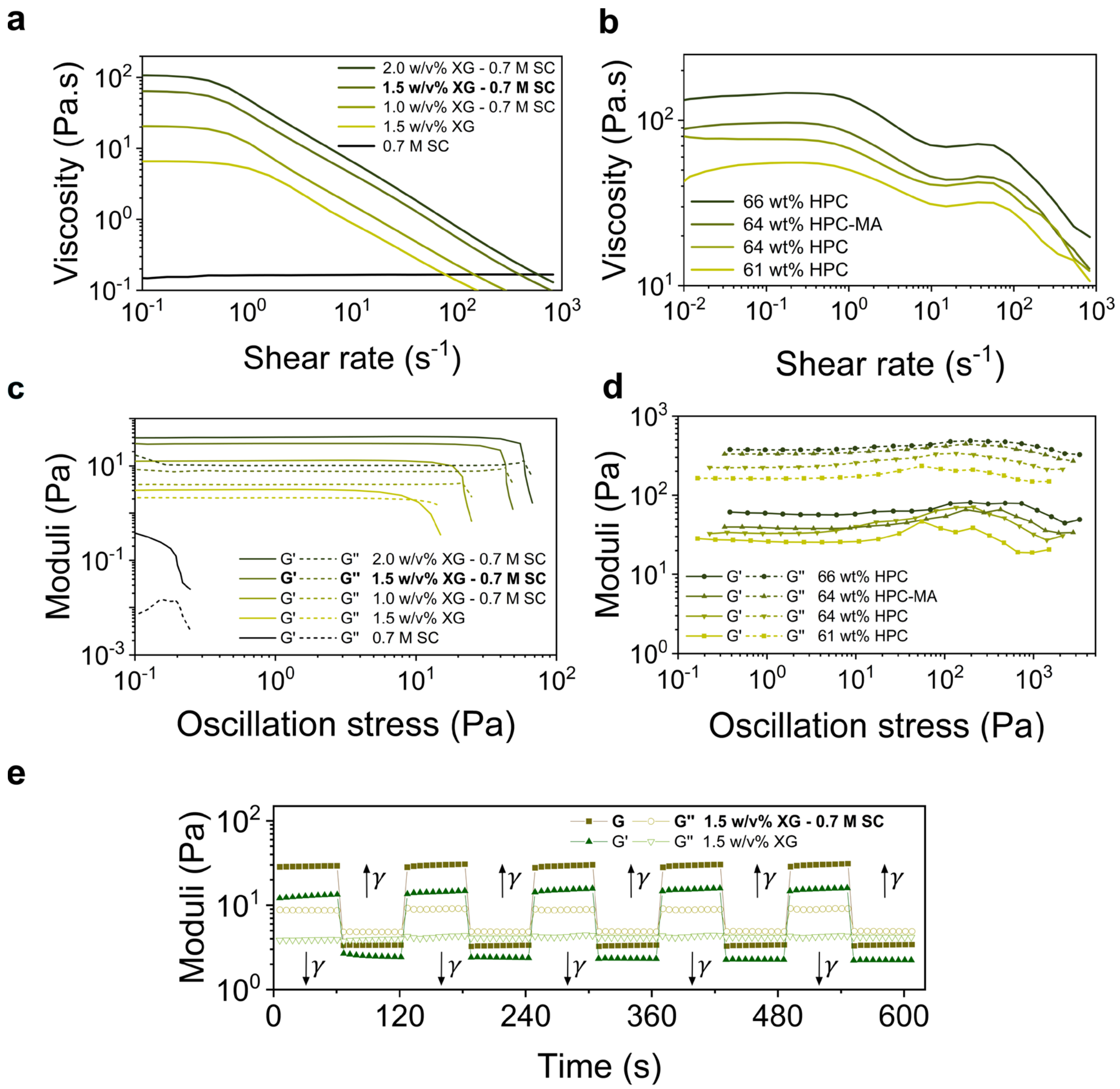

Figure S9| Rheological characterization of XG-SC support baths and HPC inks. a – b) Apparent viscosity as a function of shear rate for (a) support baths containing different XG concentrations and (b) HPC inks. **c – d)** Shear storage moduli ($G^{'}$) and loss moduli ($G^{''}$) as a function of shear stress for (c) support baths with different XG concentrations and (d) HPC inks. **e)** Three interval thixotropy tests (3ITT) for 1.5 w/v% XG – 0.7 M SC bath and 1.5 w/v% XG bath, indicating that the SC-XG bath exhibits good thixotropic recovery performance. The 3ITT test was conducted under alternating structural restoration at a low shear strain condition (amplitude strain = 1%) for 60 s, followed by structural deformation at a high shear strain condition (amplitude strain = 500%) for 60 s. The XG-SC bath can recover 90% of its storage modulus within 4 s.

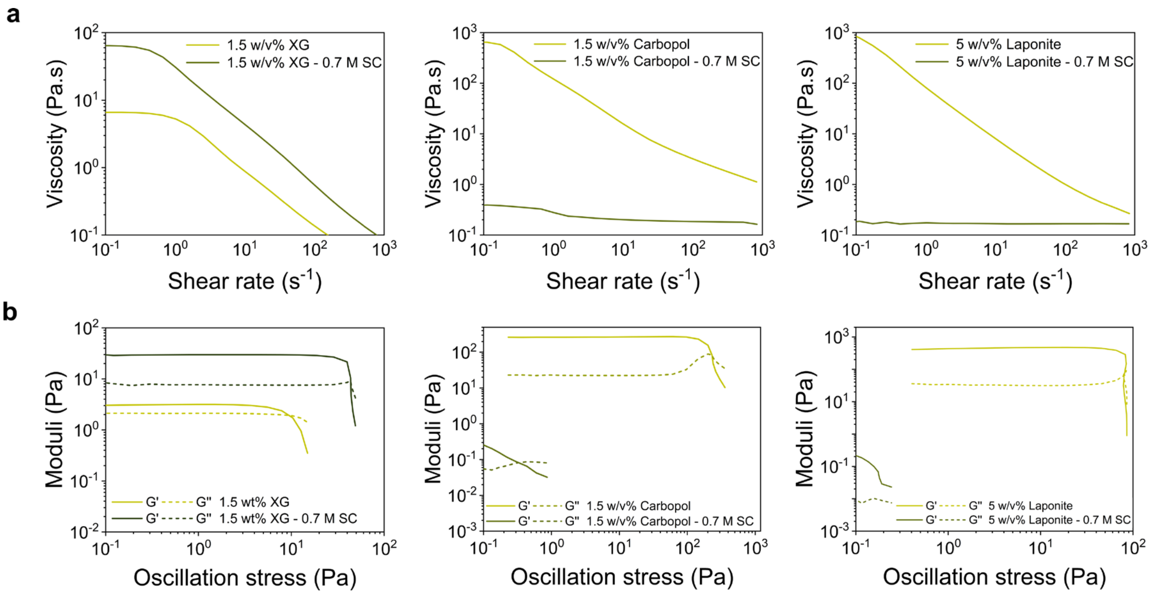

Figure S10| Effect of ionic strength on the rheology of XG and other representative supportive baths. **a)** Apparent viscosity vs shear rate for 1.5 w/v% XG, 1.5 w/v% Carbopol and 5 w/v% Laponite XLG support baths, with or without the addition of 0.7 M SC. **b)** Shear storage moduli ($G^{'}$) and loss moduli ($G^{''}$) vs shear stress for the support baths. Compared to Carbopol and Laponite support baths, the rheology of XG baths was minimally affected by the ionic strength of SC.

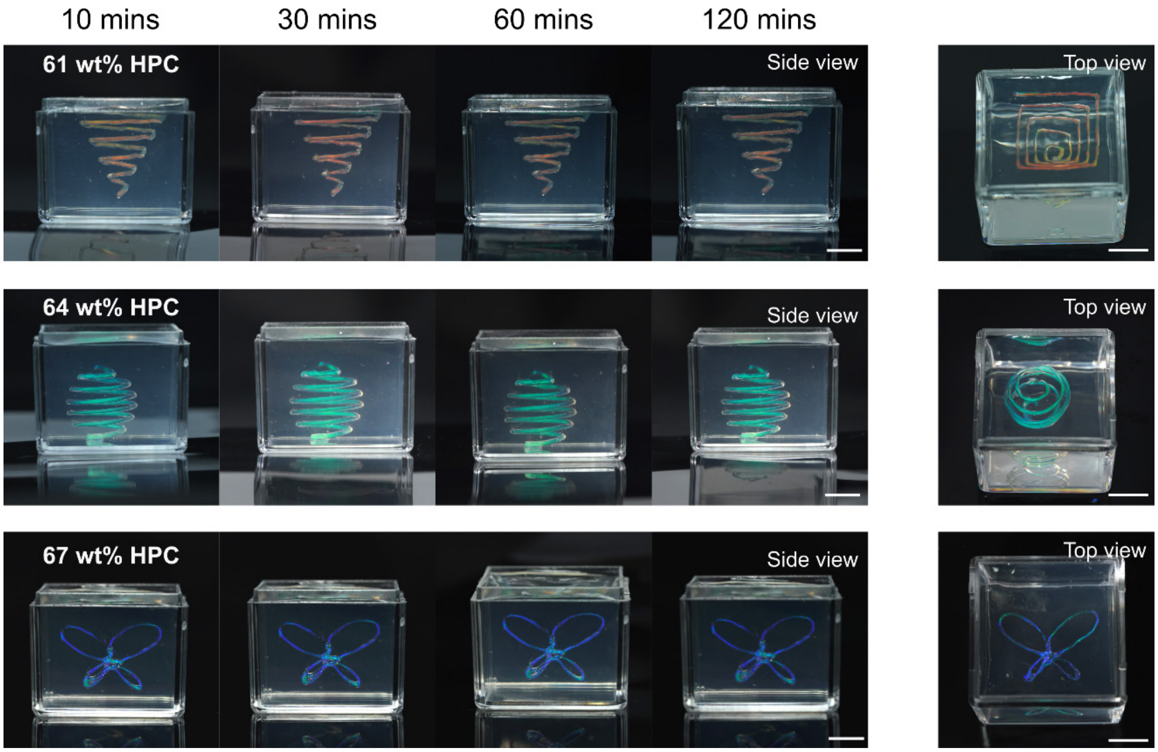

Figure S11| Stable ATPSs formed between SC-XG support baths and cholesteric HPC inks at varying concentrations. The structural colors of the HPC inks had no noticeable change over 120 mins in the baths. Scale bars, 10 mm.

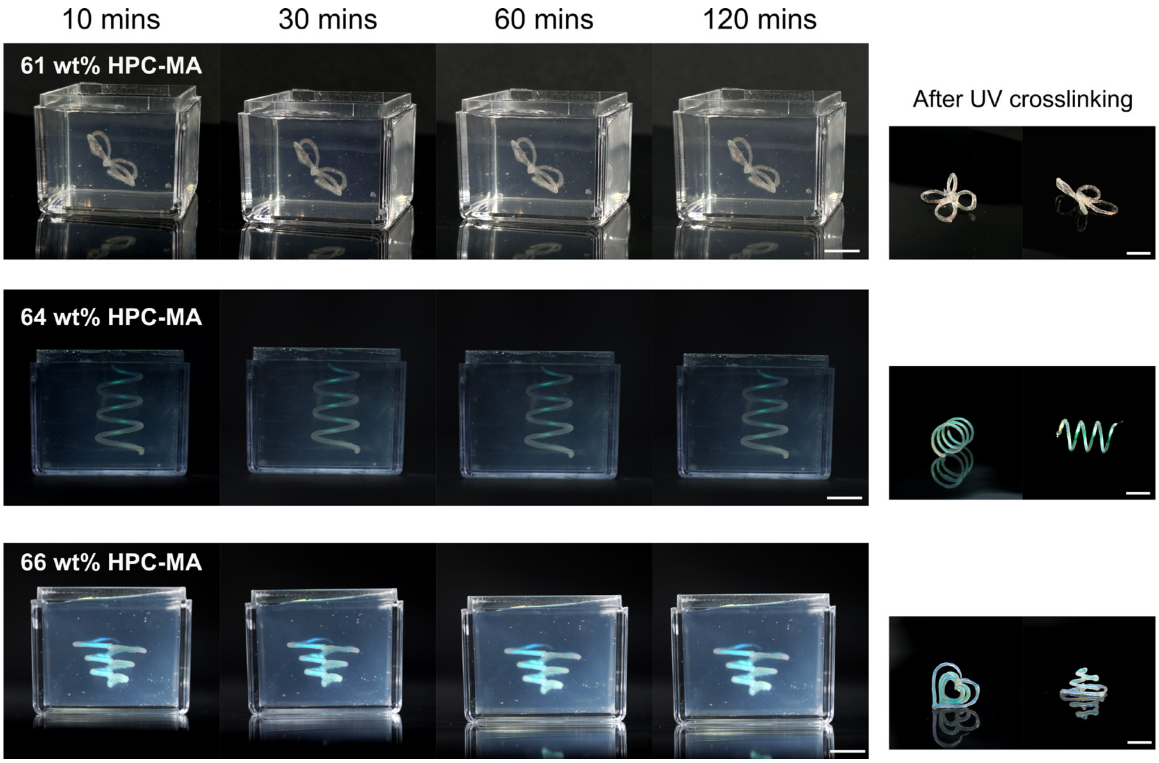

Figure S12| Stable ATPSs formed between SC-XG support baths and cholesteric HPC-MA inks at varying concentrations. The structural colors of the HPC-MA inks had no noticeable change over 120 mins in the baths. The HPC-MA inks in the bath can be UV crosslinked to obtain freeform solidified HPC photonic structures. Scale bars, 10 mm.

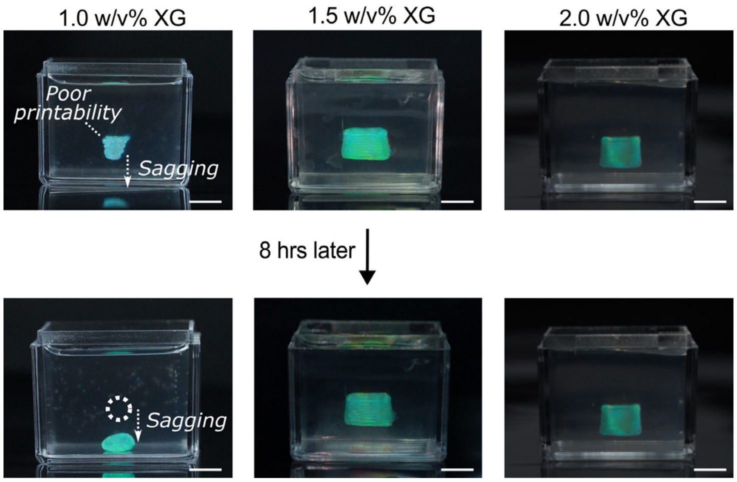

Figure S13| Photographs showing the evolution of the printed cholesteric HPC inks in the SC-XG support baths with concentrations of 1 w/v%, 1.5 w/v% and 2 w/v% XG. The 1.5 w/v% XG concentration was selected for the support baths, as the 1 w/v% XG concentration cannot provide adequate printability and mechanical properties for supporting the printed ink. The rheological measurements of the baths can be found in **Figure S9**. Scale bars, 10 mm.

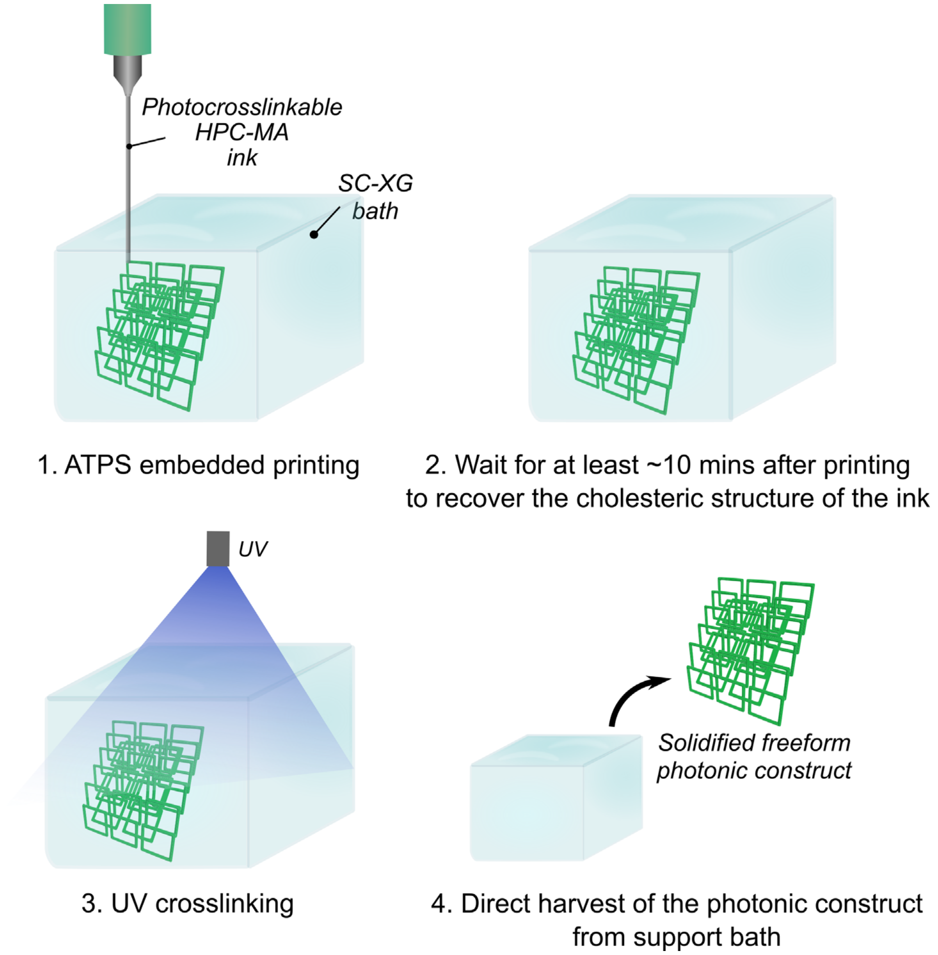

Figure S14| Schematic depicting the ATPS embedded printing procedure to manufacture solid cellulosic photonic structures.

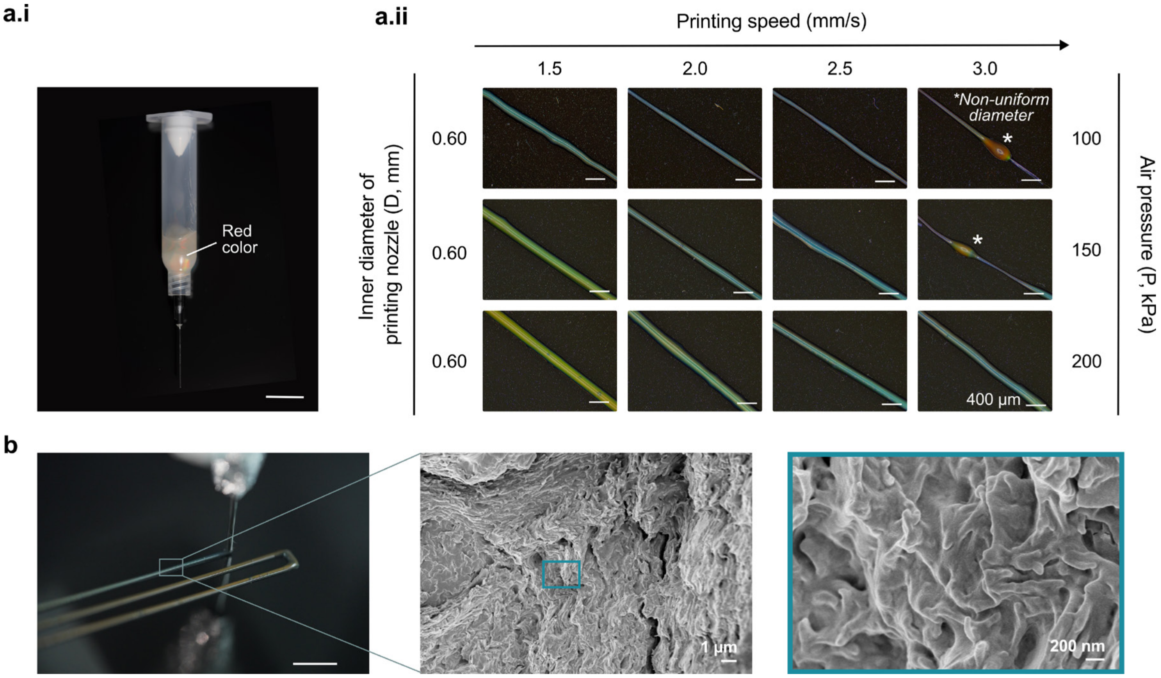

Figure S15| Limitations of in-air 3D printing for fabricating cholesteric HPC inks.

a) Changes in structural color due to water loss during the in-air 3D printing process. **(a.i)** Photograph of 61 wt% HPC-MA ink in a syringe, which exhibited a red color before extrusion. **(a.ii)** Microscopic images of the extruded filaments.

The extruded filaments in **(a.ii)** displayed a noticeable blue shift in colors compared to the ink shown in **(a.i)**. A more pronounced blue shift occurred in filaments with smaller diameters due to a more severe water loss during printing and UV crosslinking. Additionally, achieving thin filaments with uniform diameters using in-air extrusion printing is difficult due to surface tension.

b) Photograph of a 61 wt% HPC-MA filament printed via in-air extrusion printing. The SEM images were taken from the cross sections of the samples after UV crosslinking.

Immediately after extrusion, the filament exhibited a blue shift in its structural color due to the shear-induced disruption of cholesteric mesophases caused by extrusion, as evidenced by the SEM images. Thus, in situ UV crosslinking results in a substantial color loss in the printed filament. Scale bars in (a.i) and (b), 10 mm.

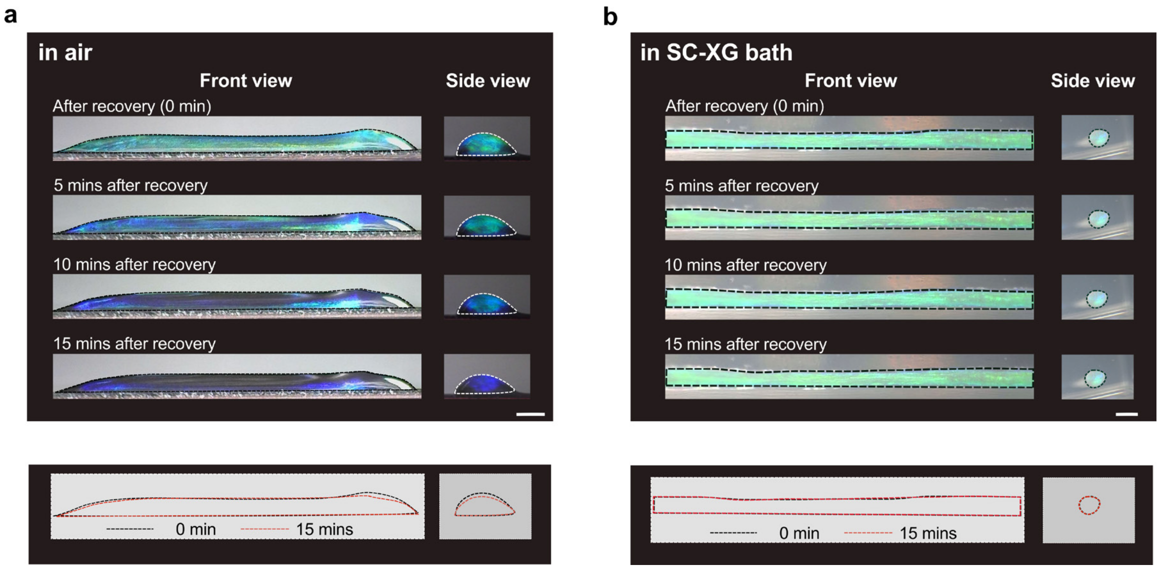

Figure S16| Evolution of the color and shape of printed filaments: a) in air and b) in SC-XG bath after recovery from shear. The filaments in air exhibited a blue-shifted color and a compressed shape over time due to water evaporation and surface tension effect, whereas the color and shape of the filament in the SC-XG bath were well-preserved. The observations were made after a recovery period of 2 mins following printing. Scale bars, 1 mm.

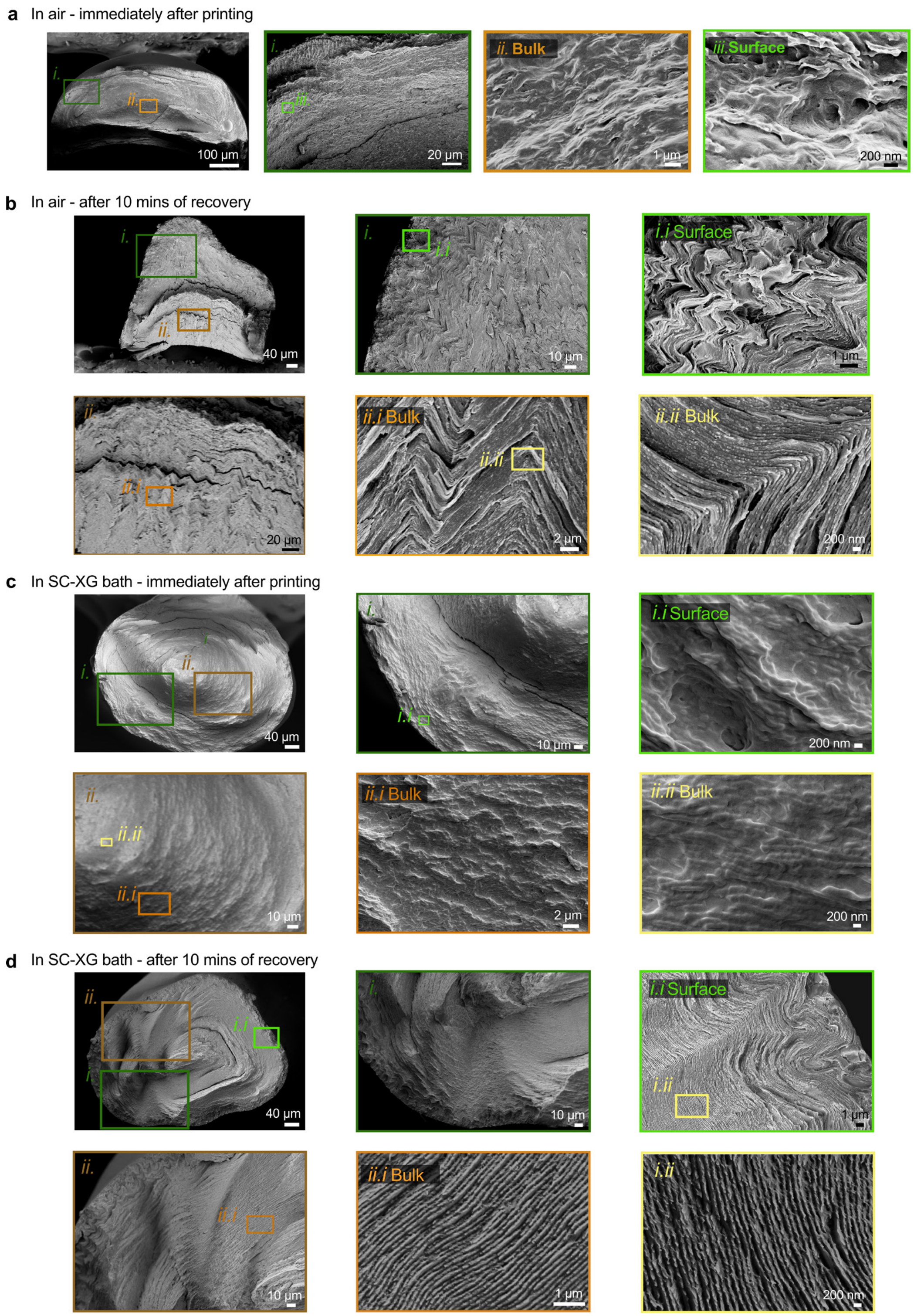

Figure S17| Microstructure analysis of the printed HPC-MA filament under different printing conditions and recovery time. a) Immediately after printing in air, **b)** after recovering for 10 mins from in-air printing, **c)** immediately after ATPS embedded printing, and **d)** after recovering for 10 mins in the bath from ATPS embedded printing.

- Compared to ATPS embedded printing, the filaments produced by in-air printing exhibited significant macroscopic deformation due to ink-air surface tension and drying-induced buckling effect during recovery.

- Without a recovery period, both in-air printing and ATPS embedded printing resulted in a highly disordered structure (**Figure S17a** and **Figure S17c**), lacking cholesteric structure at both the surface and core, which explains their non-colored appearance after printing.

- For the filament recovered for 10 minutes after in-air printing, severe wrinkling of the microstructure was observed at the surface (**Figure S17b**). This phenomenon is due to Helfrich-Hurault instabilities arising from the relaxation of the cholesteric phase after shear deformation, along with evaporation-driven compression and buckling. The bulk of the filament exhibited long-range cholesteric structures with uniform alignment. However, the cholesteric pitch was significantly compressed compared to the ink before printing, resulting in a colorless appearance (**Figure S18b**).

- For the filament recovered for 10 mins after ATPS embedded printing, less severe surface wrinkles were observed than in air printing **(Figure S17d)**. This can be attributed to the hindered evaporation effects in the bath. Long-range cholesteric structures with uniform alignment were predominantly present throughout the entire cross-section of the filament. The cholesteric structure was similar to that of the ink before printing, thereby preserving structural color (**Figure S18b**).

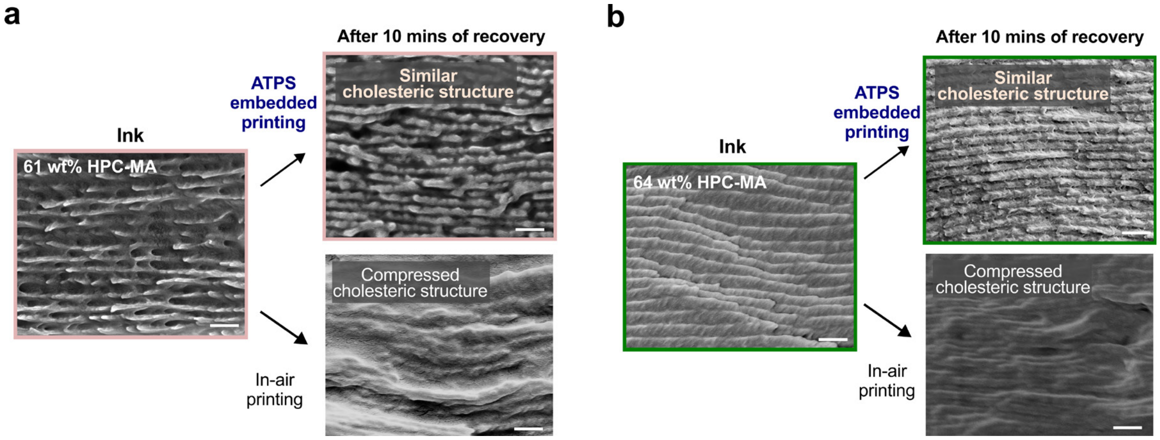

Figure S18| Recovery of the cholesteric structures of HPC-MA filaments under different printing conditions. a) 61 wt% and **b)** 64 wt% HPC-MA filaments after a 10-min recovery following ATPS embedded printing or in-air printing. All samples were UV crosslinked. Scale bars, 500 nm.

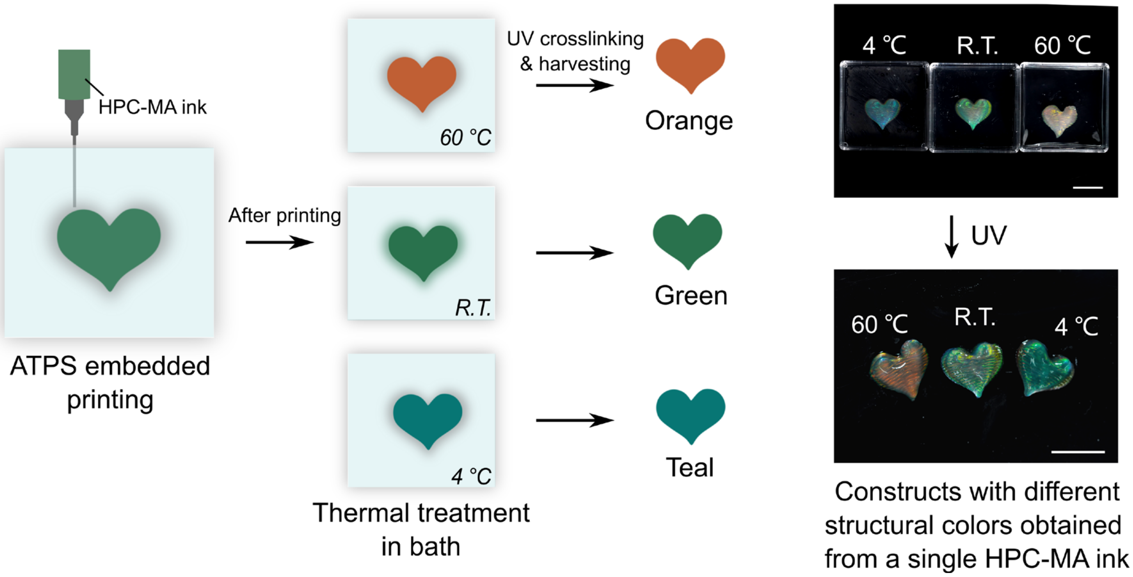

Figure S19| Constructs with various structural colors fabricated using a single HPC-MA feedstock by modulating the temperature of the bath before UV crosslinking. The bath containing the 64 wt% HPC printed structures were subjected to thermal treatment for at least 30 mins before and during the crosslinking process. During thermal treatment, the ATPSs remained stable. Warming the bath led to a red shift of the embedded construct, while cooling resulted in a blue shift. Scale bar, 10 mm. R.T. = room temperature.

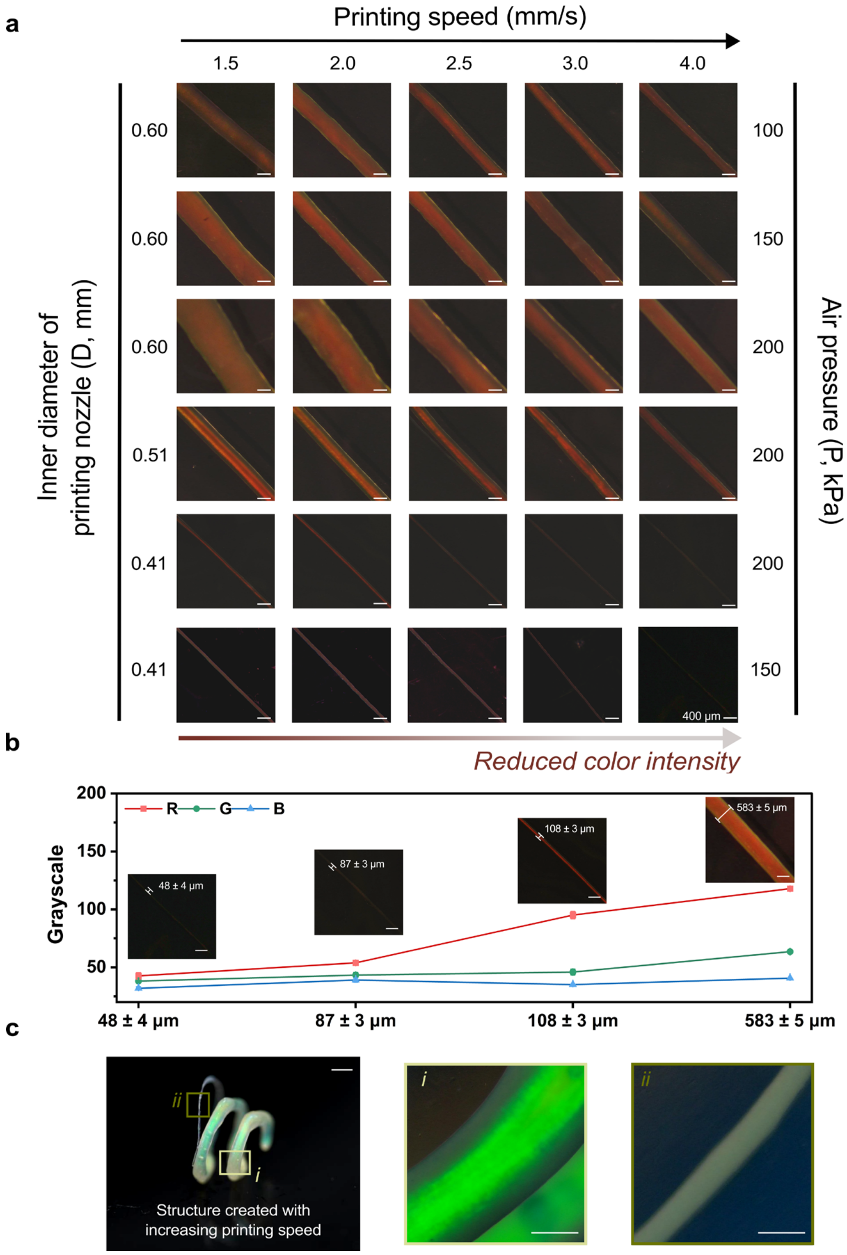

Figure S20| Adjustability of the diameters and color intensity of the extruded filaments produced using ATPS embedded printing method. a) Different filaments with uniform diameters can be achieved through modulation of the printing parameters. The structural color intensity diminished when reducing the filament diameter. The ink used here was 61 wt% HPC-MA. **b)** ImageJ analysis on the grayscale values of the red (R), green (G) and blue (B) channels of the printed filaments with different diameters shown in **Figure 4b**. A significant reduction in the grayscale values was observed when the diameter was less than 100 µm. **c)** A structure with varying thickness can be created by increasing the printing speed during printing, which allows for heterogeneous intensities of structural colors within a single construct of 64 wt% HPC-MA. Scale bars in (b) = 400 µm, (c) = 2 mm, and (c.i – c.ii) = 0.5 mm. The samples in (a-b), (c.i) and (c.ii) were immersed in a SC-XG solution during imaging to avoid surface scattering.

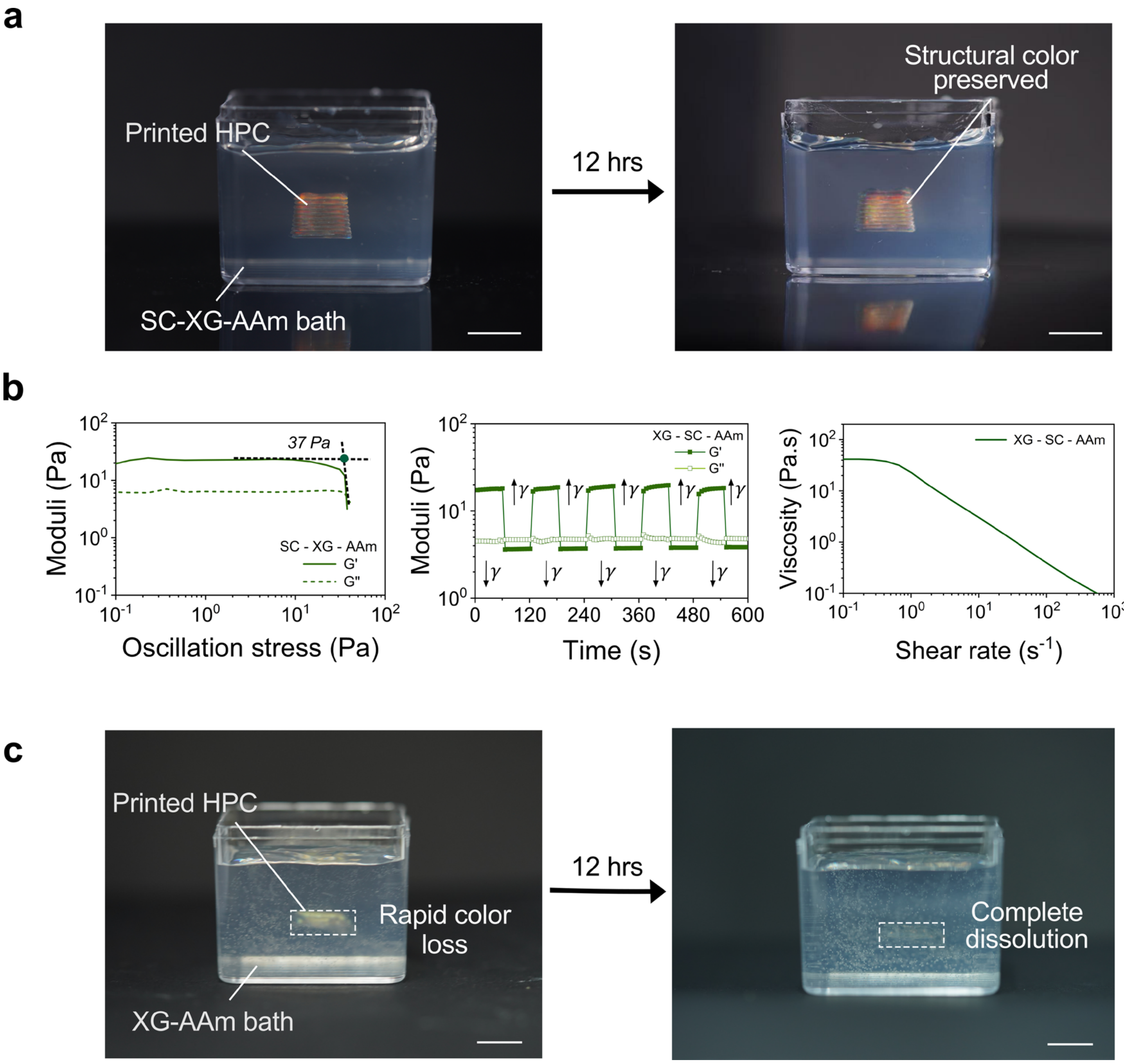

Figure S21| ATPS and rheological characterization of SC- XG-AAm matrix. a) Printed HPC in immiscible SC-XG-AAm bath. Stable ATPS can be formed with the SC-XG-AAm matrix as the salt-rich phase and a cholesteric ink of 61 wt% HPC as the polymer-rich phase. The structural colors of the embedded cholesteric HPC ink had no noticeable change over 12 hrs. **b)** Shear storage modulus (*G'*) and loss modulus (*G''*) versus oscillation stress plot, three interval thixotropy test (3ITT), and viscosity versus shear rate plot for the SC-XG-AAm bath. The 3ITT test was conducted under alternating structural restoration at a low shear strain condition (amplitude strain = 1%) for 60 s, followed by structural deformation at a high shear strain condition (amplitude strain = 500%) for 60 s. 3ITT test indicates that the SC-XG-AAm bath exhibits good thixotropic recovery performance. The XG-SC-AAm bath can recover 90% of its storage modulus within 4 s. **c)** Printed HPC in XG-AAm bath. The printed HPC rapidly lost color in the XG-AAm bath without the addition of SC. Scale bars in (a) and (c), 10 mm.

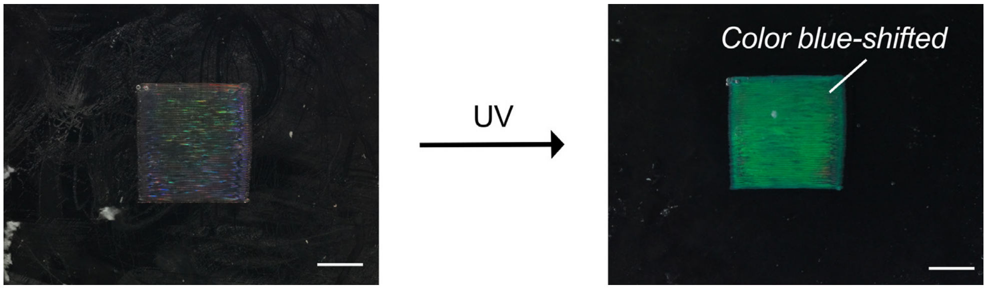

Figure S22| Structural color of the embedded HPC ink before and after UV crosslinking of the matrix. A blue shift in the cholesteric ink of 61 wt% HPC was observed after UV crosslinking attributed to the water evaporation and matrix shrinkage effects during UV exposure. Scale bar, 10 mm.

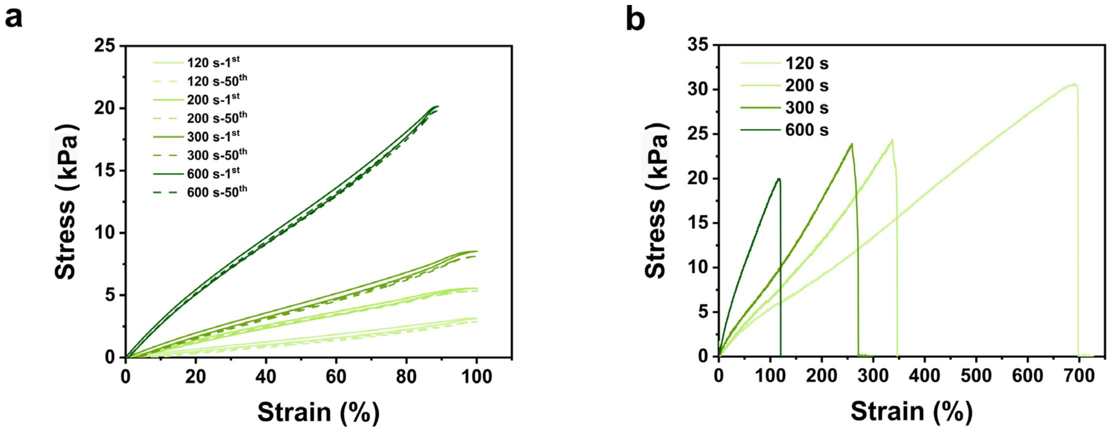

Figure S23| Tensile mechanical properties of the SC-XG-AAm matrices produced with different UV crosslinking times. a) Representative cyclic loading-unloading stress-strain curves for samples treated with different UV crosslinking times (i.e., 120 s – 600 s). **b)** Representative tensile stress-strain curves.

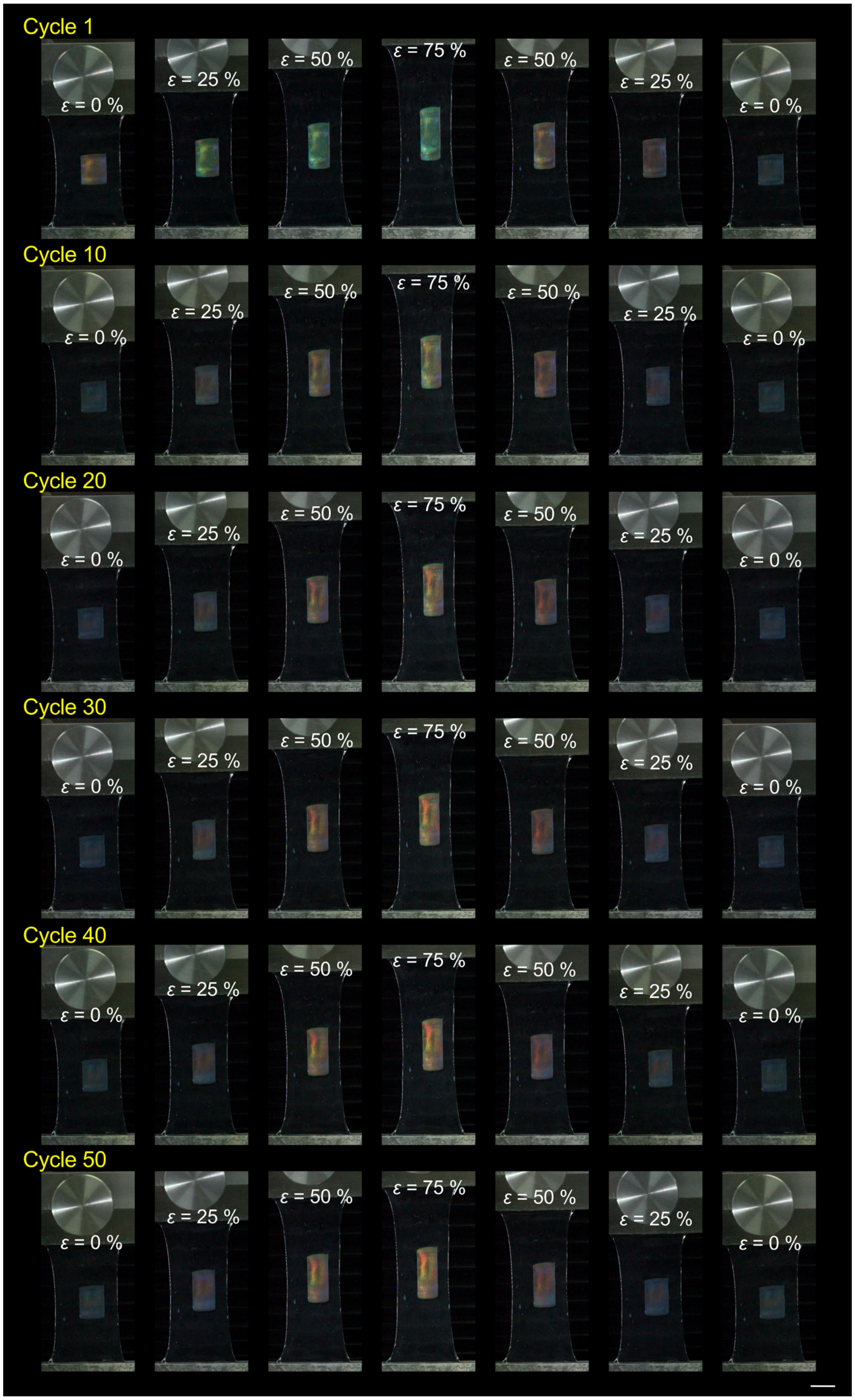

Figure S24| Mechanochromic response of a MechanoHPC hydrogel over 50 stretching cycles at a strain rate of 150 mm/min. The responses during 1st, 20^{th,} 30^{th,} 40th and 50th cycles are illustrated. The MechanoHPC hydrogel became stable after 20 cycles. Scale bar, 10 mm.

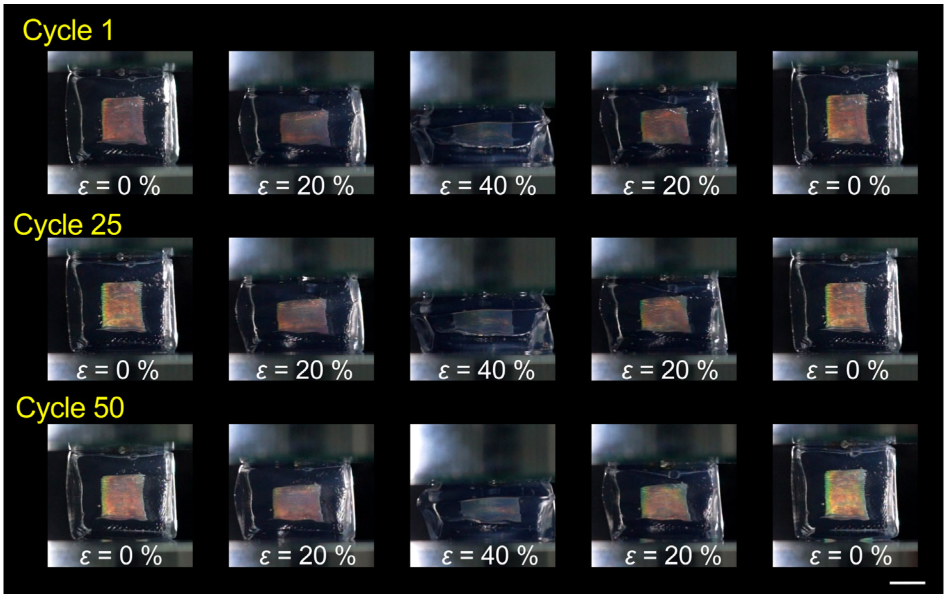

Figure S25| Compressive mechanochromic response of a MechanoHPC hydrogel over 50 compression cycles of at a strain rate of 50 mm/min. The responses during 1st, 25th and 50th cycles were illustrated, showing consistent color changes. Scale bar, 10 mm.

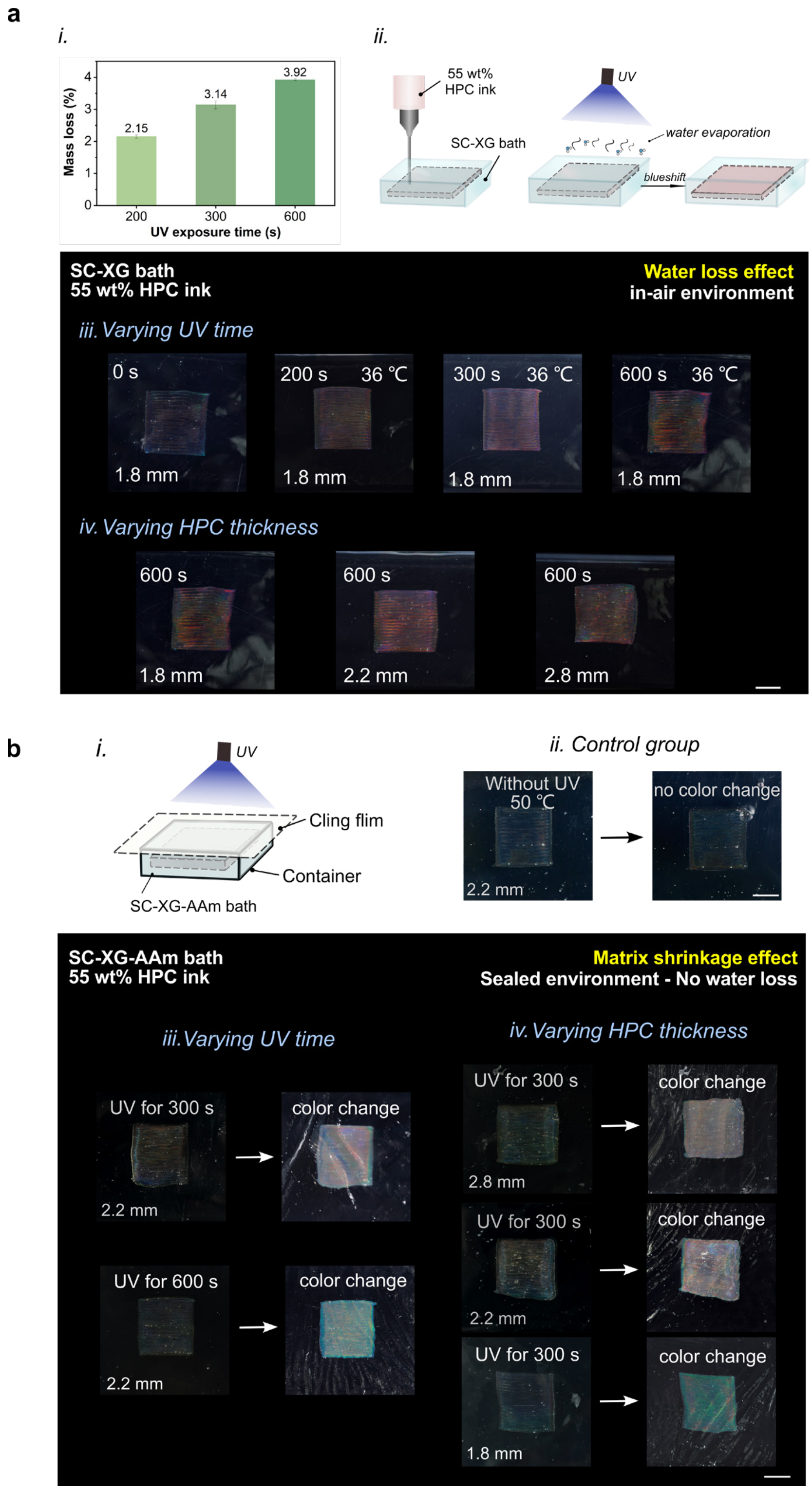

Figure S26| Effect of temperature-induced water loss and matrix shrinkage on the color of HPC during UV exposure. a) Color shifts caused by temperature-induced water loss. **(a.i)** During UV exposure of SC-XG-AAm matrices, the temperature of the UV chamber can reach ~36 ºC, leading to mass loss and water evaporation. **(a.ii)** To focus solely on water evaporation effect and

eliminate the influence of matrix shrinkage, we investigated the color changes in HPC layers printed in an uncrosslinkable SC-XG bath. **(a.iii)** The HPC films showed a blue shift with longer UV exposure, attributed to the longer temperature-induced water evaporation effect. **(a.iv)** However, varying the thickness of the embedded HPC does not lead to a noticeable color difference due to the same UV exposure duration. **b)** Color shifts caused by matrix shrinkage. **(b.i)** To examine the effect of matrix shrinkage and eliminate the water evaporation effect, a MechanoHPC device was wrapped in polyethylene cling film during UV exposure. **(b.ii)** To validate the effectiveness of this method in preventing evaporation, a control group was placed in an oven at 50 °C. No color change was observed after returning to room temperature, confirming that the cling film effectively prevents water evaporation. The effect of UV crosslinking is depicted in **(b.iii)**. A UV crosslinking time of 300 s resulted in a blue shift in HPC, changing from pale red to intense visible red. Further increases in exposure time (i.e., 600 s) blue-shifted the HPC layer to light green. **(b.iv)** The blue shift became more pronounced as the HPC thickness decreased, attributed to the increased compression effect on the embedded HPC layer. The above finding indicates that both temperature-induced water loss and matrix shrinkage resulting from UV exposure can alter the color of the MechanoHPC devices. Scale bars, 10 mm.

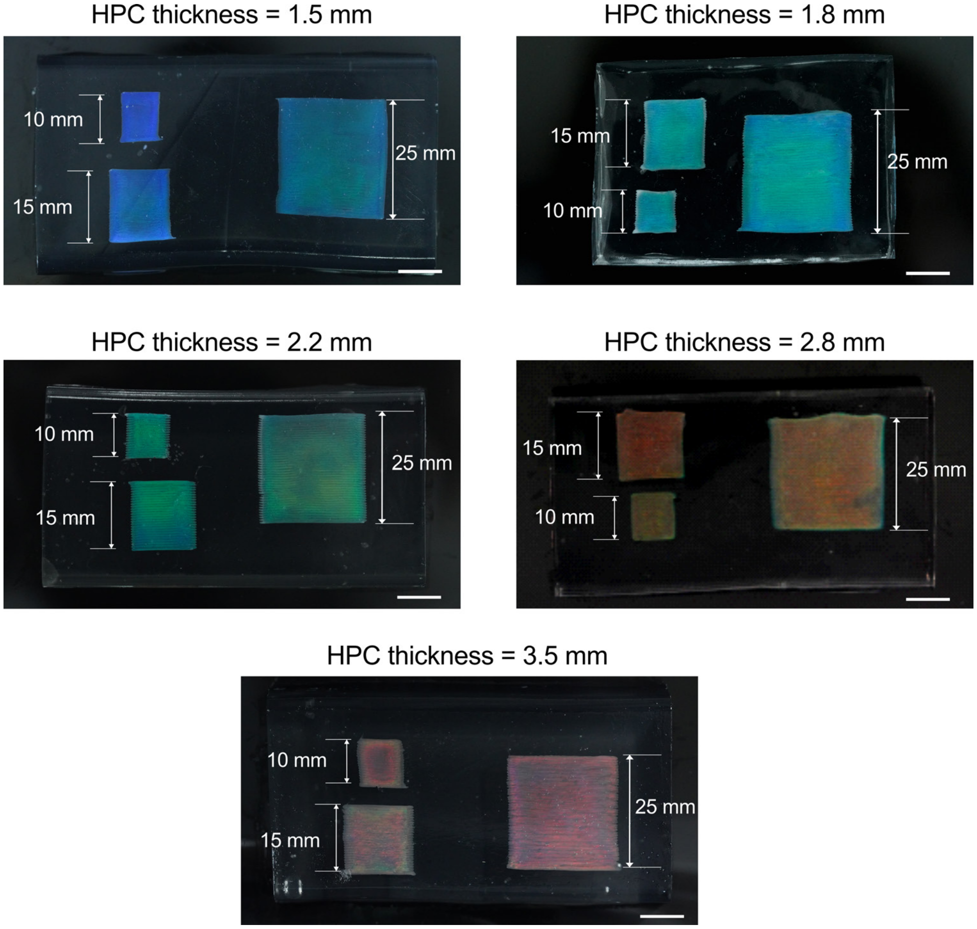

Figure S27| Effect of HPC pattern size on structural coloration. All MechanoHPC hydrogels were fabricated under a constant UV crosslinking time of 600 s using an HPC ink of 55 wt%. The length and width of the printed HPC patterns did not substantially influence its structural color, but its thickness has a crucial impact on its structural color. The thickness was measured using an optical microscope. Scale bars, 10 mm.

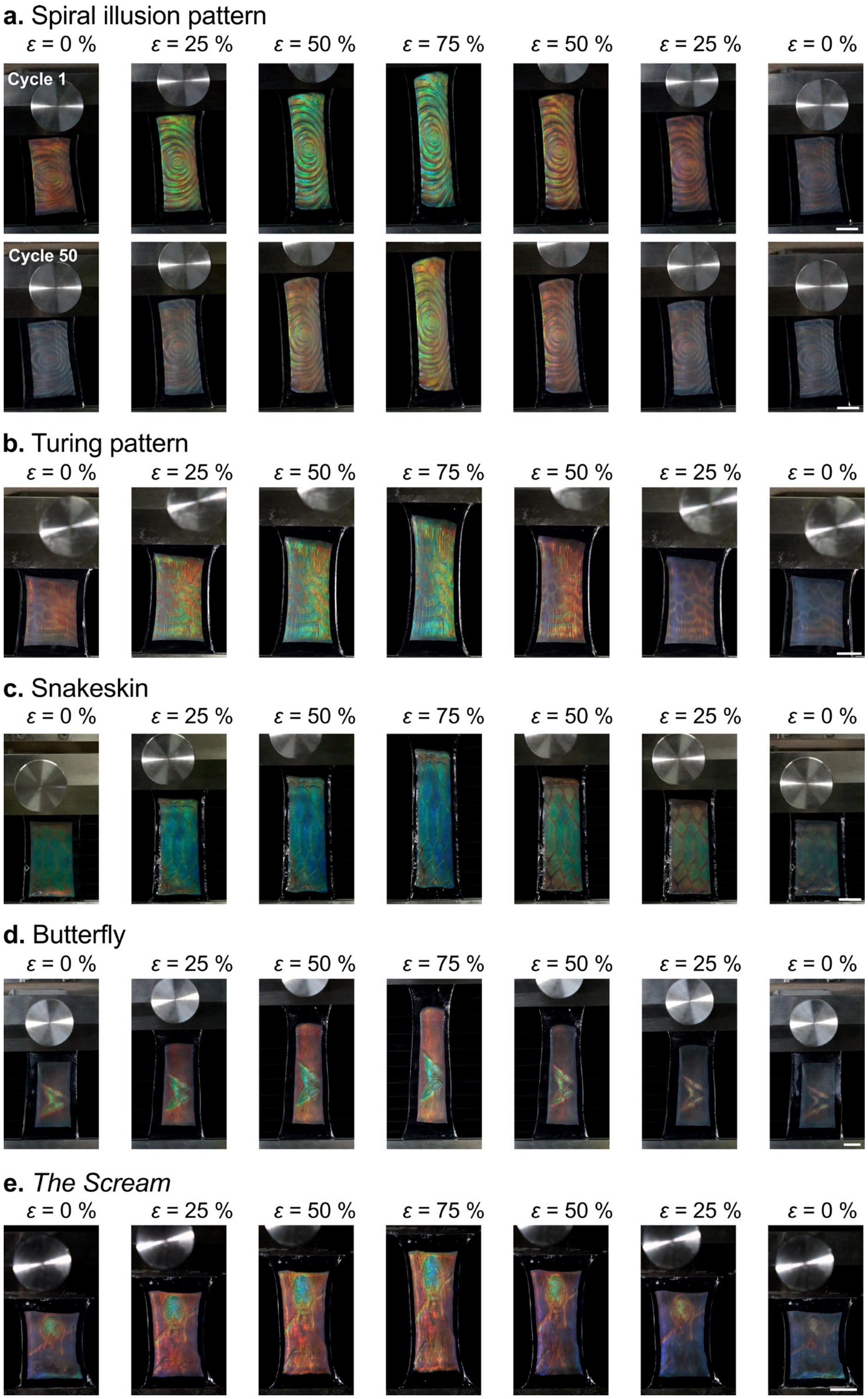

Figure S28| Mechanochromic response of the MechanoHPC hydrogels DLP-printed with intricate patterns. a) Spiral illusion pattern, **b)** Turing pattern, **c)** Snakeskin pattern, **d)** butterfly pattern and **e)** *The scream*. The samples were stretched at a strain rate of 500 mm/min. Scale bars, 10 mm.

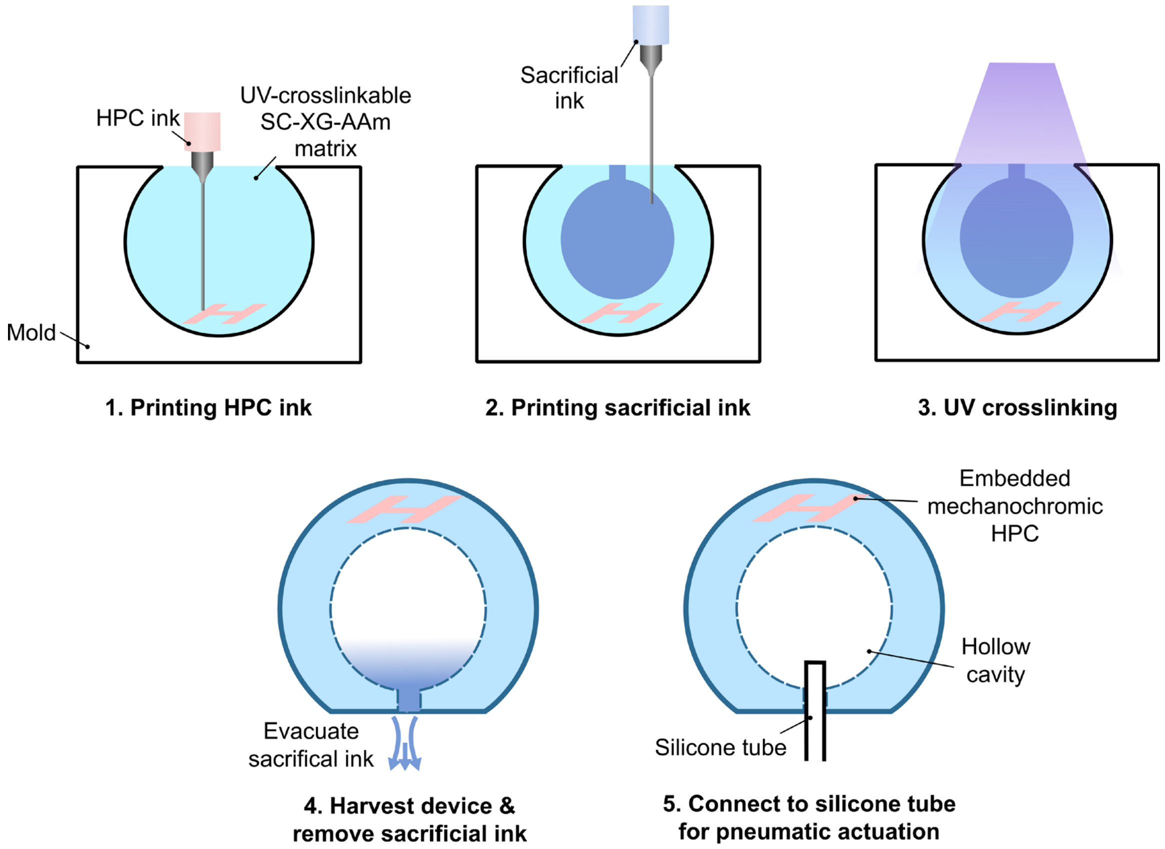

Figure S29| Schematic illustrating the fabrication of a MechanoHPC hydrogel actuator for information encryption.

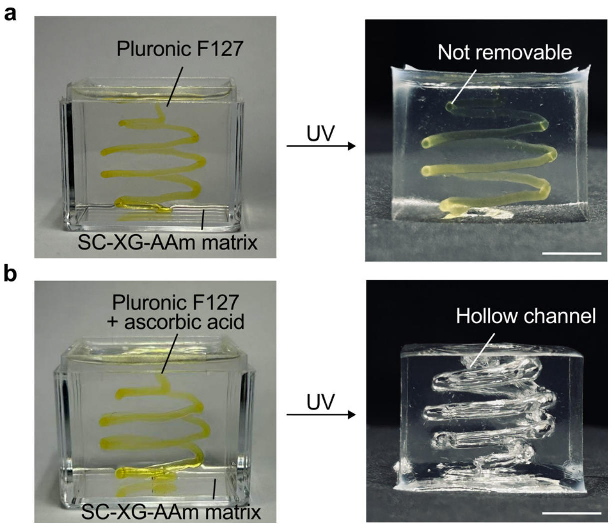

Figure S30| The effect of ascorbic acid in preventing photo-polymerization of the diffused AAm monomers in the sacrificial ink region. a) The printed sacrificial ink cannot be removed after UV crosslinking when the sacrificial ink is not supplemented with 2 w/v% ascorbic acid. **b)** Sacrificial ink can be removed when the sacrificial ink is supplemented with 2 w/v% ascorbic acid. The Pluronic F127 concentration in the sacrificial ink was 35 w/v%, and all samples were UV crosslinked for 200 s. Scale bars, 10 mm.

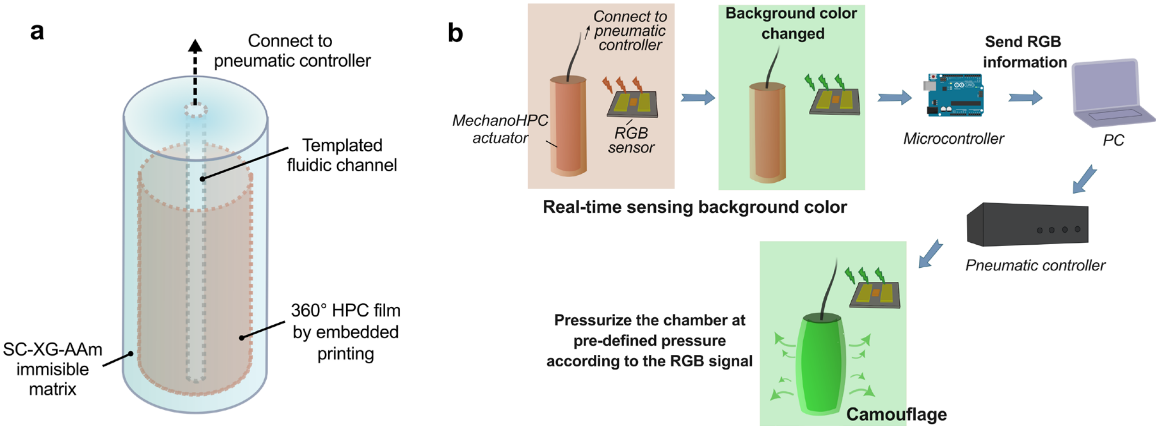

Figure S31| Schematic depicting a) the design and b) the control logic of a cylindrical MechanoHPC actuator for camouflage.

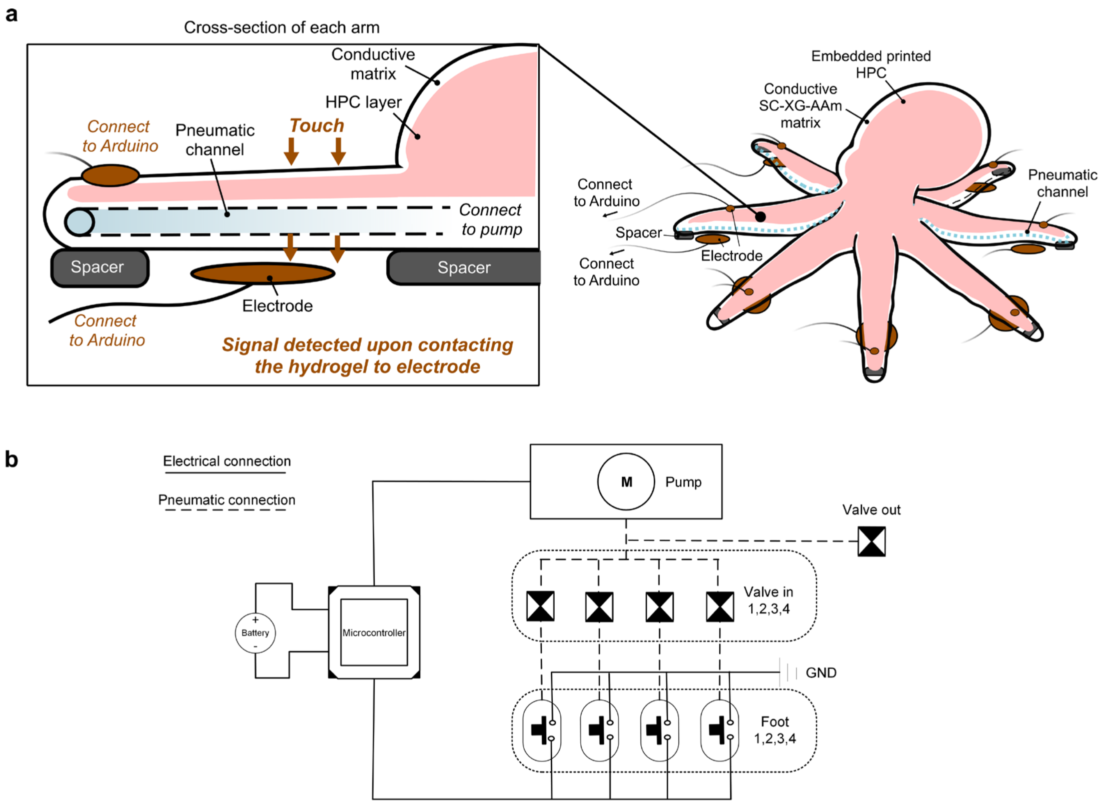

Figure S32| Schematic depicting a) the design and b) the control logic of the MechanoHPC octopus actuator, which possesses tactile sensing and human-robot interaction capabilities. This design employs spacers below the arms. Electrodes were placed on each arm and situated below the arm. When each arm of the octopus actuator was touched, a signal was generated and detected by an Arduino, which subsequently controlled the pump to pressurize the arm in response to the touch stimuli. Each arm can be controlled independently.

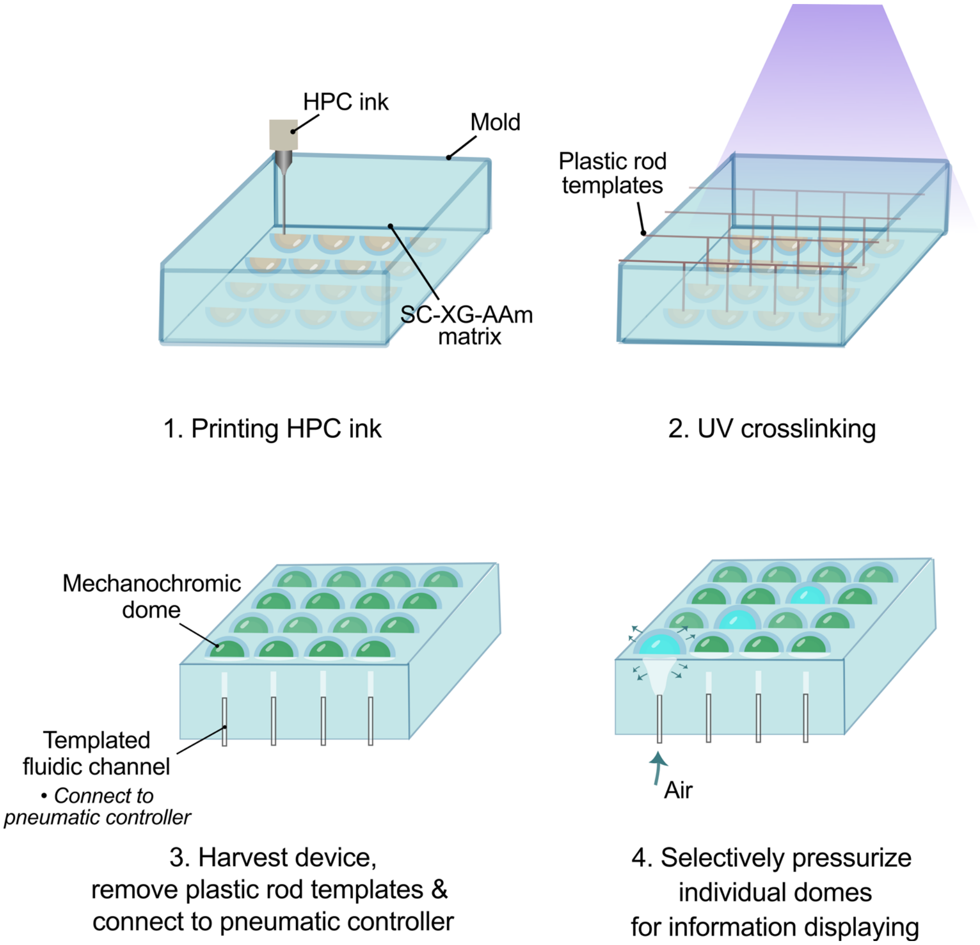

Figure S33| Schematic depicting the fabrication of the MechanoHPC hydrogel display.

Movie S1| Embedded printing process of HPC in a support bath composed of sodium citrate and xanthan gum.

Movie S2| Mechanochromic responses of MechanoHPC hydrogels under stretching at strain rates of 150 mm/min and 500 mm/min. 50 cycles of stretching at 75% strain were performed.

Movie S3| Mechanochromic response of a MechanoHPC hydrogel under compression. 50 cycles of compression at 40 % strain were performed at a strain rate of 50 mm/min.

Movie S4| Multicolored MechanoHPC hydrogels embedded printed with "UM" and "C" patterns under stretching.

Movie S5| Multicolored MechanoHPC hydrogels DLP-printed with intricate patterns. The hydrogels were stretched at 75 % strain over 8 cycles.

Movie S6| A MechanoHPC actuator for information encryption.

Movie S7| Background matching ability of a cylindrical MechanoHPC hydrogel actuator.

Movie S8| A biomimetic MechanoHPC octopus actuator with tactile perception and human-robot interaction capabilities.

Movie S9| A Programmable mechanochromic display based on MechanoHPC Hydrogels.

Supplementary references

[1] B. E. Droguet, H.-L. Liang, B. Frka-Petesic, R. M. Parker, M. F. L. De Volder, J. J. Baumberg, S. Vignolini, *Nat. Mater.* **2022**, *21*, 352.

[2] R. M. Parker, T. H. Zhao, B. Frka-Petesic, S. Vignolini, *Nat. Commun.* **2022**, *13*, 3378.

[3] J. Song, R. M. Parker, B. Frka-Petesic, T. Deng, L. Xu, X. Deng, S. Vignolini, Q. Shen, **n.d.**, DOI 10.1002/adma.202416607.

[4] C. A. Williams, R. M. Parker, A. Kyriacou, M. Murace, S. Vignolini, **n.d.**, DOI 10.1002/adma.202307563.

[5] H.-L. Liang, M. M. Bay, R. Vadrucci, C. H. Barty-King, J. Peng, J. J. Baumberg, M. F. L. De Volder, S. Vignolini, *Nat. Commun.* **2018**, *9*, 4632.

[6] Q. Wang, C. Wang, Z. Fang, Z. Zhang, Y. Zhao, T. Ma, L. Shang, *Adv. Sci. Weinh. Baden-Wurtt. Ger.* **2025**, *12*, e06556.

[7] Q. Wang, Z. Zhang, C. Wang, X. Yang, Z. Fang, L. Shang, *Adv. Sci.* **2024**, *11*, 2308442.

[8] X. Ma, B. Wu, L. Hou, P. Wu, *ACS Nano* **2025**, *19*, 23945.

[9] K. George, M. Esmaeili, J. Wang, N. Taheri-Qazvini, A. Abbaspourrad, M. Sadati, *Proc. Natl. Acad. Sci.* **2023**, *120*, e2220032120.

[10] C. L. C. Chan, I. M. Lei, G. T. van de Kerkhof, R. M. Parker, K. D. Richards, R. C. Evans, Y. Y. S. Huang, S. Vignolini, *Adv. Funct. Mater.* **2022**, *32*, 2108566.

[11] Z. Zhang, C. Wang, Q. Wang, Y. Zhao, L. Shang, *Proc. Natl. Acad. Sci.* **2022**, *119*, e2204113119.
